# Nanoplastic toxicity and uptake in kidney cells: differential effects of concentration, particle size, and polymer type

**DOI:** 10.1007/s10565-025-10135-2

**Published:** 2026-01-16

**Authors:** Hayden Louis Gillings, Darling M. Rojas-Canales, Soon Wei Wong, Kaustubh R. Bhuskute, Amandeep Kaur, Iliana Delcheva, Jonathan M. Gleadle, Melanie MacGregor

**Affiliations:** 1 Flinders Institute for Nanoscale Science and Technology, College of Science and Engineering, Flinders University, Sturt Road, Bedford Park, South Australia 5042 Australia; 2 Flinders Health and Medical Research Institute, College of Medicine and Public Health, Flinders University, Sturt Road, Bedford Park, South Australia 5042 Australia; 3 Nano and Microplastics Research Consortium, Flinders University, Sturt Road, Bedford Park, South Australia 5042 Australia; 4 Department of Renal Medicine, Flinders Medical Centre, Flinders Drive, Bedford Park, South Australia 5042 Australia; 5 Medicinal Chemistry, Monash Institute of Pharmaceutical Sciences, Monash University, Parkville, Melbourne, VIC 3052 Australia; 6 Australian Research Council Centre of Excellence for Innovations in Peptide and Protein Science, Monash University, Melbourne, VIC 3052 Australia

**Keywords:** Nanoplastics, Kidney, Cytotoxicity, Polystyrene, Poly(methyl methacrylate), Polyethylene

## Abstract

Nanoplastics (NPs, < 1 µm) are emerging environmental contaminants capable of crossing biological barriers and interacting at the cellular and subcellular level. Despite evidence of microplastics in human kidney tissue and urine, the renal effects of NPs remain poorly understood. This study investigated the short-term effects of NPs polymer type, size, and concentration on human kidney proximal tubule cells (HK-2). Cells were exposed for 24-h to carboxylated polystyrene (PS), poly(methyl methacrylate) (PMMA), and polyethylene (PE) NPs (15–100 nm) at concentrations from 0.1 to 200 µg/mL. NPs morphology, size, and charge were characterised by scanning electron microscopy, dynamic light scattering, and zeta potential. Cell morphology, viability, cell cycle distribution, and NPs internalisation were assessed by microscopy and flow cytometry. Low-concentration exposures had minimal effects, whereas 100 and 200 µg/mL induced marked morphological changes, including cytoplasmic granularity. Viability decreased significantly at 200 µg/mL for several NPs types, with PE NPs causing the largest reduction (79.4%). Polymer type influenced outcomes, with PE and PMMA NPs causing greater morphological disruption than PS. Size effects were most evident in cell cycle analysis: 15 nm and 20 nm PS NPs and 100 nm PMMA NPs induced phase arrest without major viability loss. NPs internalisation increased with concentration but varied with polymer type, with PE NPs showing preferential perinuclear localisation. These findings demonstrate that NPs effects on kidney cells depend on polymer chemistry, particle size, concentration, and highlight the need for long-term studies using environmentally relevant NPs to better assess kidney toxicity risk.

**Supplementary Information:**

The online version contains supplementary material available at 10.1007/s10565-025-10135-2.

## Introduction

In recent years, there has been increasing concern regarding the health risks and the potential toxicity associated with exposure to nanoplastics (NPs). NPs are typically defined as any plastic particles below 1 µm in diameter, either intentionally manufactured (e.g. latex nanoparticles) or the byproduct of plastic degradation in the environment (International Organization for Standardization 2020). They represent the smallest size fraction of microplastics (MPs) which are defined as plastic fragments smaller than 5 mm. It is important to note that particles that are 1 µm in diameter differ significantly from 100 nm NPs in their behaviour and properties due to factors such as Brownian motion, diffraction limits and enhanced ability to cross cell membrane (Atugoda et al. 2022; Gigault et al. 2021; Masseroni et al. 2022). At this point in time, it is unknown at what rate MPs are degrading to NPs, the concentrations at which NPs persist within the environment, nor the full extent of the harm they pose when they enter biological systems (Thompson et al. 2024). These uncertainties are made more difficult to resolve by the diversity of polymers and the presence of additives, such as plasticisers, colourants and stabilisers, which all contribute to the complexity of detecting and assessing NPs within the environment (Monclús et al. 2025).

While the majority of reports of the effects of plastic particle exposure have focused on larger size fractions, there are compelling reasons to investigate the effects of nanoparticles below 100 nm — particularly regarding their behaviour in biological matrices and potential health impacts — as particles in this size range can more readily cross biological barriers, interact at the cellular and subcellular level, and exhibit unique surface reactivity (Lamoree et al. 2025). So far research into the toxicity and potential health effects of NPs has been predominantly focused on the lungs, liver and gastrointestinal system (Kihara et al. 2021; Lin et al. 2022; Schwarzfischer et al. 2022), with minimal examination of the impact and toxicity of NPs on the renal system (Goodman et al. 2022). This is particularly concerning as MPs have been detected within a wide range of human tissues and biofluids, with lung tissue and blood being some of the first reports (Lamoree et al. 2025; Leslie et al. 2022). More recently, MPs have recently been detected in the arterial plaque of patients with carotid artery disease (Marfella et al. 2024), and healthy human kidney tissue and urine samples (Marfella et al. 2024; Massardo et al. 2024; Nihart et al. 2025). Massardo et al. (2024) found 26 MPs between 1 and 29 µm in kidney tissue, and between 3 and 13 µm in urine samples. Nihart et al. (2025) assessed kidney tissue samples from 2016 and 2024 and reported a total plastic mass of 402.5 and 404.8 µg per gram of tissue, respectively. This study also noted an increased incidence of MPs within the kidney tissue localised to the glomeruli and tubules.

Given these findings, it is important to understand how this accumulation may be occurring within the renal system. The kidney filters about 180 L of blood each day, removing waste while reclaiming essential substances like electrolytes, glucose, and proteins (Molitoris et al. 2022; Zhuo and Li 2013). This process begins in the glomerulus, where blood is filtered. The glomerular filtrate then continues into the renal tubules, where most solutes are reabsorbed, particularly in the proximal tubule, which handles up to 80% of this workload (Adhipandito et al. 2021; Zhuo and Li 2013). The glomerular filtration barrier (GFB) is composed of podocytes, glomerular endothelial cells and the glomerular basement membrane (Nielsen et al. 2016). The GFB is theorised to have a pore size of 4 nm (Adhipandito et al. 2021), and yet a known filtration limit of 6–8 nm, while albumin, the most common protein within blood, has a diameter of 14 nm at its widest point and 3.8 nm at its smallest point (Gburek et al. 2021; Molitoris et al. 2022). As a result, only small amounts of albumin pass through the GFB under normal conditions, but through the use of specialised endocytic pathways facilitated by podocytes, they are then reabsorbed by tubular cells and recycled (Gburek et al. 2021). Importantly, these same pathways may also allow nano-sized particles, such as NPs, to cross the barrier and be reabsorbed by tubular cells, providing a possible explanation for their accumulation within the kidney.

Previous nanoparticle exposure studies have observed accumulation of particles in both the GFB and proximal tubule, and in some cases, complete filtration of the particles into urine (Fan et al. 2021; Lawrence et al. 2017; Naumenko et al. 2019; Williams et al. 2018; Zuckerman and Davis 2013). In these studies, the charge of the particles was also addressed, concluding that positively and negatively charged particles differed in their accumulation behaviour. Specifically, due to the negative charge of the GFB, positively charged nanoparticles had a higher propensity to accumulate, compared to their negatively charged counterparts (Naumenko et al. 2019; Zuckerman and Davis 2013). Other studies have highlighted differences in cytotoxicity, inflammation and cell response following exposure to various nanoparticles, such as silver (Kennedy et al. 2019), zinc oxide (Shehata et al. 2022), and silica (Passagne et al. 2012; Rafieepour et al. 2019; Wang et al. 2009). This indicates that biological outcomes may be driven not only by the particle physical property (nanosize and shape) but also by their chemical properties, such as surface charge, atomic composition and functional groups, which influences interactions with cell membranes, organelles and biomolecules. We hypothesise that NPs cytotoxicity and accumulation within the kidney differ depending on their polymer type, size, surface chemistry and charge. Yet, most studies evaluating the toxicity of NPs on mammalian cells only look at 1 type of polymer: polystyrene (PS). Across current NPs studies, PS NPs are overrepresented – despite accounting for only ~ 6% of global plastic waste—while other polymer types remain largely understudied (Pradel et al. 2023). This narrow focus limits both environmental relevance and toxicological insight, as different polymers may engage in substantially different interactions with biological systems. Expanding NPs research to include a broader range of polymer types and sizes is therefore necessary to better reflect real-world exposures and uncover polymer-specific toxicological effects.

In this study we used PS, poly(methyl methacrylate) (PMMA) and polyethylene (PE) NPs because these polymers are among the few available commercially as monodisperse NPs in reproducible, affordable batches suitable for systematic experimentation. While PS and PMMA are not the most abundant polymers in environmental litter, their widespread use in model studies has generated a substantial comparative literature, and PE remains one of the most prevalent plastics in the environment. All particles were purchased with carboxyl (–COOH) surface functionalisation, as this modification provides more reliable suspension behaviour than unmodified NPs, which tend to aggregate and sediment (Gambinossi et al. 2015). Although environmental NPs display more heterogeneous surface chemistries, their interactions in natural waters are largely governed by the formation of protein and organic coronas, which often screens intrinsic surface charge (Alimi et al. 2022; Simpson et al. 2025). The use of COOH-modified particles therefore provides a controlled and widely accepted starting point for probing polymer-dependent differences in behaviour.

Given the potential for accumulation, recirculation and filtration of NPs via the kidney, testing the fate and effects of small NPs, particularly those less than 100 nm, is crucial to understanding renal impact. The proximal tubules play an essential physiological role of internalising nanoscale particles and proteins through receptor-mediated endocytosis, pinocytosis and transcytosis. In this study, the proximal tubule cell line, Human Kidney 2 (HK-2), was therefore selected to assess renal cell uptake and toxicity of NPs. HK-2 cells were exposed to PS, PMMA, and PE NPs, from 15 to 100 nm in size, at concentrations ranging from 0.1 µg/mL to 200 µg/mL. It is hypothesised that exposure to higher NPs concentrations will result in increased cell response, while cell response will vary dependent on the polymer type.

The toxicity and biological effects of NPs with varying sizes, polymers and concentrations on HK-2 cells was assessed, with the aim of evaluating how these factors impact cell viability, cell cycles and internalisation and to provide insight into the potential implications of short-term NPs exposure on kidney function.

## Materials and methods

### Nanoplastics

Carboxylated (-COOH) NPs of various sizes and polymer types were obtained from multiple suppliers. Specifically, PS NPs 20 nm (PS20-A) and 100 nm (PS100-A) in diameter were purchased from Thermo Fisher Scientific (Manufacturer A). PS NPs 15 nm (PS15-B) and 100 nm (PS100-B) in diameter, 50 nm PMMA (PMMA50-B) and PE (PE50-B) NPs were purchased from Lab261 (Manufacturer B). PMMA NPs 50 nm (PMMA50-C) and 100 nm (PMMA100-C) in diameter were purchased from Phosphorex (Manufacturer C). For NPs internalisation experiments, different types of fluorescent NPs were used. An initial experiment was performed to test for potential fluorophore leaching effects, using both internally and externally labelled particles: externally labelled red fluorescent (RF) 100 nm PS NPs (Magsphere Inc), and 100 nm Fluoro-Max PS NPs (Thermo Fisher Scientific) internally labelled with Europium (Eu). Eu, as a fluorophore, can be beneficial for bioaccumulation studies due to its large Stokes shift, and reduced susceptibility to photobleaching, which in turn can reduce the effects of autofluorescence and increase visibility of NPs (Cheignon et al. 2022; Crawford et al. 2015). For subsequent internalisation experiments, fluorescently labelled PS15-B-RF, PS100-B-RF, PMMA50-B-RF and PE50-B-RF were used to test the effect of polymer type on internalisation.

The PS20-A and PS100-A NPs were provided respectively as 41 mg/mL and 45 mg/mL stock solutions in deionised water. All NPs from manufacturers B and C were supplied as 10 mg/mL stock solutions in deionised water containing surfactant and 2 mM sodium azide (NaN_3_). The volume and type of surfactants used in the stock solutions were unknown aside from those supplied by Lab261, which were specified to be 0.1% Tween-20.

#### Dilutions

Immediately prior to dilution, all NPs stocks were briefly vortexed and then sonicated in a water bath sonicator (Soniclean Digital Benchtop Ultrasonic Cleaner 80TD) for 15 min to facilitate homogenisation of the NPs suspensions. All non-fluorescent NPs were diluted to concentrations of 2 mg/mL (2,000 µg/mL), 200 µg/mL, 20 µg/mL and 2 µg/mL in 1 × Phosphate Buffered Saline (PBS) (Gibco). All fluorescent NPs were diluted in PBS to concentrations of 1 mg/mL (1,000 µg/mL), 100 µg/mL, 10 µg/mL and 1 µg/mL, or in the case of the RF and Eu PS NPs, these were diluted to the same number of particles per cell as the non-fluorescent 100 nm PS NPs to ensure consistency with the NPs exposure experiments. Prior to cell treatment, all NPs dilutions were further diluted at a ratio of 1:10 into cell culture media to final concentrations of 200 µg/mL, 20 µg/mL, 2 µg/mL and 0.2 µg/mL for NPs exposure experiments, or 100 µg/mL, 10 µg/mL, 1 µg/mL and 0.1 µg/mL for NPs internalisation experiments. The particles per mL concentrations of the NPs stock solutions were either provided by the manufacturer upon receipt of the stocks, or calculated using the following equation:

1 $$particles\;per\;mL=\frac{grams\;per\;mL}{density\times(4/3\times\pi r^3)}$$

To determine the number of particles per cell during the exposure period, the following calculation was used:

2 $$particles\;per\;cell=\frac{(particles\;per\;mL\times well\;volume)}{number\;of\;cells}$$

A full summary of the NPs suspensions used in this study can be found in Table S1 and S2.

Additionally, potential particle delivery to the cell surface during the 24-h exposure was estimated using an established in-vitro dosimetry model (DeLoid et al. 2015; NovaMechanics Ltd n.d.). Parameters derived from the dynamic light scattering (DLS) measurements in treatment media (cell culture medium + 10% PBS) were used as model input as detailed in Table S3.

#### Characterisation

Attenuated total reflectance (ATR) with Fourier transformed infrared spectroscopy (FTIR) was used to confirm the chemical composition of the NPs. Characterisation was performed on a Thermo Fisher spectrometer Nicolet iS50 with a DuraScope ATR attachment with ZnSe crystal. Background and sample spectra were collected at 64 number of scans with 2 cm^−1^ resolution at spectral range 600 to 4000 cm^−1^. The shape, size, polydispersity index (PDI) and surface charge of all NPs used was assessed via scanning electron microscopy (SEM), DLS and zeta potential measurement, respectively. DLS analysis was performed using NPs suspensions at 100 µg/mL prepared in Milli-Q water, PBS, and cell culture media to mimic the cell experiment conditions. For SEM, a 5 µL drop of the suspension in MilliQ water was placed on a silicon wafer and allowed to air dry before coating the substrate with a thin layer of platinum to prevent beam damage. Imaging was conducted with a Gemini II (Bruker), equipped with a field emission gun and operated at an accelerating voltage of 5 kV and a working distance of 3 mm. For the smallest NPs (PS15-B, and PS20-A), as well as PE50-B, ultra high-resolution scanning electron microscope (Hitachi High-Tech Co. Model SU8600) equipped with cold-cathode field emission gun operated at 1 kV was used. The secondary electron (SE) images were taken with in-column SE detector. DLS measurements were carried out on a Zetasizer Nano ZS (Malvern Instruments, UK) with a 4 mW He–Ne laser (633 nm) as a light source and a non-invasive backscatter (NIBS) detection angle of 173°. The analysis was performed at 25 °C in disposable PS cuvettes with 2 min sample equilibration. The same equipment in conjunction with a folded capillary cell under Smoluchowski approximation was used for zeta potential measurements. Each sample was measured in triplicate.

### Cell culture and nanoplastics exposure

HK-2 cells (ATCC CRL-2190) were cultured in 1:1 Dulbecco’s Modified Eagle Medium (DMEM) and Ham’s F-12 (Gibco), and supplemented with 10 mM HEPES (Gibco), 10% fetal calf serum (FCS; Bovogen Biologicals) and 1X penicillin, streptomycin and glutamine mix (Gibco) at 37 °C with 5% CO_2_. Cells were seeded at 1 × 10^5^ cells/well in 12-well cell culture plates, or at 1 × 10^4^ cells/well in 96-well clear bottom cell culture plates, and incubated for 24-h before replenishing the cells with control or NPs treatment media at volumes of 500 µL for 12-well plates, or 100 µL for 96-well plates. The NPs concentrations in different well volumes utilised across the experiments were set to ensure consistent nanoplastic particle-per-cell numbers within the experiments and are summarised in Table S1 and S2. The HK-2 cells were incubated with the control or NPs treatment media for 24-h before proceeding with the intended assay.

### Cell morphology assessment

For experiments using standard NPs, cells were imaged after the 24-h NPs incubation period, immediately prior to cell collection. Images were taken using a Thermo Fisher EVOS M5000 Imaging System, or a ZEISS Primovert inverted cell culture microscope with a ZEISS Axiocam 208 color/202 mono microscope camera. Micrograph images were taken of cells at the centre of the plate wells, with a 40 × objective, immediately prior to commencement of the live/dead cell viability assay and cell cycle analysis outlined below.

Cell segmentation and quantitative morphology analysis were performed using QuPath (v0.5.1) (Bankhead et al. 2017) in combination with a Cellpose v4 (Cellpose-SAM) (Pachitariu et al. 2025) model to ensure accurate delineation of individual HK-2 cells following nanoparticle exposure. Segmentation was conducted on 40 × brightfield micrographs acquired after the 24-h treatment period. Cellpose-SAM segmentation was implemented within QuPath to avoid reliance on threshold-based, nucleus-seeded identification. The model was applied at a detection resolution of 0.5 µm per pixel with a tile size of 2048 pixels and an overlap of 256 pixels to accommodate large fields of view and prevent stitching errors. Automatically generated boundaries were imported into the QuPath object hierarchy, and any cells touching the perimeter of the parent annotation region were removed to avoid partial-cell artefacts.

Following segmentation, QuPath was used to extract per-cell intensity and textural measurements from each identified cell region. Basic features, including mean, median, minimum, and maximum pixel intensity, were collected to characterise changes in intracellular signal distribution. To assess nanoparticle-induced structural alterations, texture-based Haralick features were computed within QuPath using the software’s built-in grey-level co-occurrence matrix workflow. The analysis was performed using a preferred pixel size of 0.2 µm, a 25 µm tile diameter, and grey-level normalisation between 0 and 255 intensity values, consistent with the settings illustrated in the QuPath panel. Haralick entropy was used to quantify the degree of intracellular irregularity, whereby higher values reflect increased disorder in pixel intensities, consistent with disrupted cellular organisation. Haralick difference-of-variance, referred to in the manuscript as heterogeneity, was used to measure the contrast between local pixel values across the cytoplasm, providing a quantitative index of granularity resulting from nanoparticle internalisation and cytoplasmic accumulation. These measurements were exported from QuPath and normalised to the region-of-interest (ROI) area before statistical analysis.

### Live/dead cell viability assay

A flow cytometry viability assay was performed with a modified version of the protocol provided by Thermo Fisher Scientific (2016). The HK-2 cells were washed with PBS and detached using TrypLE (Gibco) as per the manufacturer’s instructions. The detached cells, along with the supernatant and PBS washes, were collected into Fluorescence-Activated Cell Sorting (FACS) tubes and centrifuged for 5 min at 300 × g, then washed and resuspended in 100 µL of PBS. A positive control containing live and dead cells (50:50) was generated by heat-treating 50 µL of the cell suspension at 65 °C for 5 min and then cooled on ice for an additional 5 min, before being transferred back to the FACS tube. All cells were washed and resuspended with PBS once again before a 30-min incubation with 0.5 µL of LIVE/DEAD Aqua dye in 500 µL of PBS, followed by a 15-min fixation with 4% formaldehyde. After fixation, the cells were centrifuged for 5 min at 300 × g before resuspension in 200 µL of PBS with 1% FCS. The stained and fixed cells were stored at 4 °C for a maximum 1–2 days prior to analysis via flow cytometry (CytoFlex-S, Beckman Coulter). Acquired flow cytometry data was processed using FlowJo (v10.8.1; BD Biosciences).

### Cell cycle analysis

HK-2 cells were detached using TrypLE as per manufacturer's instructions. All cells were centrifuged for 5 min at 300 × g, cell pellet was washed in and resuspended in 200 µL of PBS. The cells were then fixed with 100% ethanol (EtOH), diluted to a final concentration of 80%, and stored at –20 °C for a minimum of 24-h. Post fixation, cells were centrifuged for 5 min at 300 × g, and the cells were permeabilised and stained with propidium iodide (PI) staining solution. The PI staining solution consists of 200 µg/mL of RNASE-A, 50 µg/mL of PI, and 0.1% Triton X-100 diluted in PBS. Cells were incubated in the dark 30 min, and then immediately analysed via flow cytometry (CytoFlex-S, Beckman Coulter). Acquired flow cytometry data was processed using FlowJo (v10.8.1; BD Biosciences).

### Nanoplastic internalisation

Nanoplastic internalisation in HK-2 cells was assessed via fluorescence and confocal microscopy, as well as flow cytometry analysis using fluorescently labelled NPs. A full summary of the concentrations and NPs used can be found in Table S2.

#### Microscopy analysis

Post-exposure with fluorescent NPs, internalisation was assessed via fluorescence microscopy following a modified version of the methods outlined in the study by Wang et al. (2022). Briefly, the cells were washed 3 times with PBS to remove as many non-internalised NPs as possible. The cells were fixed with 4% formaldehyde for 15 min, permeabilised with 0.1% Triton X-100 for 5 min, then stained with ActinGreen 488 ReadyProbes (Alexa Fluor 488, Invitrogen) and NucBlue Live ReadyProbes (Hoechst 33342, Invitrogen) for 30 min prior to visualisation via microscopy (Nikon Eclipse Ti2; 40 × objective). All wells were imaged with the DAPI, FITC and TexasRed filter cubes, with the exception of the Eu PS NPs, which was additionally imaged with a modified Cy-5 filter cube, containing the DAPI emission filter, to ensure correct excitation, emission and visualisation of the NPs. A minimum of 3 wells were used as replicates of the cell exposure conditions, with at least 1 image per well, corresponding to an area of 192,466 µm^2^. After capture, each image was processed via NIS-Elements AR (v5.30.07; Nikon) to remove any background fluorescence, then analysed to collect the mean fluorescence index (MFI) of each filter capture.

#### Confocal imaging

Cells were imaged using Leica SP8 inverted confocal microscope (Leica Microsystems, Wetzlar, Germany) with a 63 × oil immersion objective. Hoechst 33,342, ActinGreen 488, and Nanoplastics (Red-Fluorescent PE50-B-RF) were excited using 405, 488, and 561 nm lasers, respectively. Images were acquired in the specified acquisition windows for Hoechst 33,342 (410–490 nm), ActinGreen 488 (501–550 nm), and Nanoplastics (566–730 nm). Sequential scan with the specified lasers was used to avoid spectral overlap and bleedthrough. Raw images were processed using ImageJ software.

#### Flow cytometry fluorescence detection

Following a modified version of the method outlined by Liu et al. (2022), post-exposure, the cells were washed 3 times with PBS to remove as many non-internalised NPs as possible, then detached using TrypLE as per the manufacturer's instructions. All cells were centrifuged for 5 min at 300 × g, the cell pellet was washed in PBS and fixed with 4% formaldehyde for 15 min. Following fixation, all cells were centrifuged for 5 min at 300 × g, cell pellet was washed in and resuspended in 200 µL of PBS for storage at 4 °C for a maximum 1–2 days prior to analysis via flow cytometry (CytoFlex-S, Beckman Coulter). The internalised NPs were detected using the APC channel to detect RF PS NPs, and the Violet660 channel to detect the Eu PS NPs. Acquired flow cytometry data was processed using FlowJo (v10.8.1; BD Biosciences).

### Statistical analysis

For the cell morphology, viability and cell cycle assays, three biological replicates (three wells per treatment group) were analysed per experiment, and three independent experiments were repeated on separate days. For the NPs internalisation assays, the experiment was repeated on separate days with comparable qualitative outcomes, and the quantitative analysis reported here derives from one representative experimental replicate in which three biological replicates (three wells per treatment group) were acquired, and five images per well were collected as technical replicates to generate the MFI values. For the cell morphology data, statistical significance was determined via Wilcoxon rank-sum test with Benjamini and Hochberg (BH) adjustment, using R (v.4.4.2) (R Core Team 2024). The statistical analysis of the cell viability, cell cycle analyses and NPs internalisation data was performed using GraphPad Prism (v10.4.0; GraphPad Software). Significance was determined via two-way analysis of variance (ANOVA) with multiple comparisons Uncorrected Fisher’s least-significant difference test or via unpaired one-tailed T-test.

## Results

### Nanoplastic characterisation

**Fig. 1:**
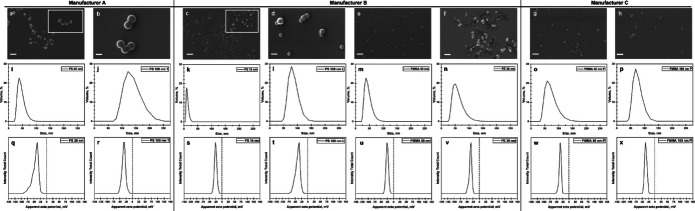
Characterisation of NPs via scanning electron microscopy (SEM). **a**) PS20-A, **b**) PS100-A, **c**) PS15-B, **d**) PS100-B, **e**) PMMA50-B, **f**) PE50-B, **g**) PMMA50-C, **h**) PMMA100-C. Scale bar = 100 nm. **i**-**p**) Size confirmation of NPs via dynamic light scattering (DLS). **q**-**x**) Zeta potential of NPs via DLS

All collected FTIR spectra matched well with the respective reference polymer materials (Fig. S1-S3), and displayed the expected key absorption peaks for PS, PMMA, and PE (Smith 2023, 2021a, 2021b, 1999). The sharp doublet at ~ 719 cm^−1^ and ~ 729 cm^−1^ in the PE50-B spectrum (Fig. S3) suggests these NPs are HDPE. Due to peak overlapping in the spectra of NPs-B, the presence of Tween surfactant was most evident in the spectrum of PE50-B (Fig. S3) (Ortiz-Tafoya and Tecante 2018). The shape, size and surface charge of the NPs was determined through SEM and DLS analysis. Visualisation of the NPs under SEM confirmed consistent size and sphericity of all NPs types investigated (Fig. 1a-h). DLS analysis confirmed that the measured hydrodynamic diameters were within the expected size range, although the degree of agreement with manufacturer reported values varied across suppliers (Table 1). Specifically, the Lab261 NPs (manufacturer B) were within 3 to 6 nm of the size indicated on their product certificate of analysis, while the Thermo Fisher particles (manufacturer A) showed larger deviations from their nominal 20 and 100 nm sizes, although the two batches still formed clearly distinct size populations. In contrast, the two Phosphorex preparations exhibited very similar hydrodynamic diameters, albeit with non-overlapping standard deviations. All particles displayed negative zeta potentials, as expected for NPs with -COOH surface modifications in Milli-Q water (Table 1). DLS measurements performed in treatment media (cell culture media + 10% PBS) showed the expected protein-associated peak at < 10 nm, together with NPs peaks whose relative intensities varied by polymer type (Fig. S4). In this medium the NPs peaks were generally broader than in PBS and less consistent between consecutive measurements, as expected in the presence of a dynamic protein corona. Additional larger-size peaks were observed for PS15-B and for both PMMA50-C and PMMA100-C samples, which may indicate some degree of aggregation. Due to strong double-layer screening at high ionic strength in exposure media, the electrophoretic mobility signals were below the instrument’s detection threshold, therefore no reliable ζ-potentials could be determined for the NPs in these conditions. Particle deposition to the cell layer over 24-h was estimated using the same media-based DLS parameters in an in-vitro dosimetry model. The calculated deposition fractions and corresponding model inputs are provided in Fig. S5 and Table S3, respectively. These estimates indicate minimal deposition for all nanoparticle types except those that displayed evidence of aggregation in treatment media.

**Table 1:** Characterisation of size and zeta potential of NPs via DLS analysis

Supplier	Polymer	PDI	Manufacturer Label* (nm)	Manufacturer Reported Diameter* (nm)	Hydrodynamic Diameter^†^ (nm)	Zeta Potential (mV)
**Thermo Fisher Scientific** **(A)**	PS	0.064 ± 0.008	20	28	48.0 ± 2.6	−41.9 ± 7.4
		0.042 ± 0.010	100	110	131.8 ± 0.6	−35.2 ± 3.2
**Lab261** **(B)**	PS	0.284 ± 0.010	15	12.5	15.6 ± 0.4	−26.9 ± 6.4
		0.052 ± 0.013	100	89	82.9 ± 1.0	−40.6 ± 3.0
	PMMA	0.077 ± 0.008	50	53	46.9 ± 0.1	−27.4 ± 2.4
	PE	0.107 ± 0.007	50	68.2	64.1 ± 0.5	−39.8 ± 4.4
**Phosphorex** **(C)**	PMMA	0.077 ± 0.012	50	50	74.9 ± 0.5	−34.9 ± 0.6
		0.056 ± 0.016	100	100	79.4 ± 0.9	−36.0 ± 0.8

*As provided by manufacturers. †As measured by DLS

### Cell morphology assessment

**Fig. 2:**
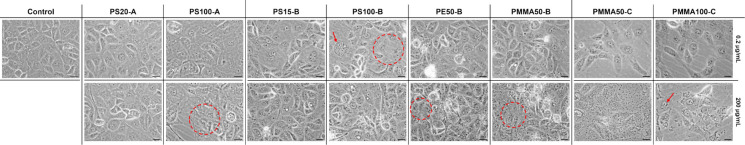
Micrographs of HK-2 cells following 24-h exposure to NPs of various sizes and polymers from three different manufacturers. All cells were treated with either PS20-A, PS100-A, PS15-B, PS100-B, PE50-B, PMMA50-B, PMMA50-C and PMMA100-C NPs at increasing concentrations from 0.2 µg/mL to 200 µg/mL. Arrows indicate cell multinucleation. Dashed lines indicate regions of undefined cell borders. 40 × magnification. Scale bar = 25 µm. N = 3. Data are representative images of replicate experiments. Representative micrographs of cells exposed to intermediate NP concentrations are provided in Fig. S6 and S7

Following the 24-h NPs exposure, changes to the morphology of the cells were assessed by microscopy analysis. When comparing cell exposure to NPs of varying sizes, polymers and concentrations, these factors could be attributed to identifiable morphological changes (Fig. 2, S6 and S7). Commonly, incidences of multinucleation increased in cells exposed to NPs across all types and sizes as compared to the control group and more so in the lower 0.2 µg/mL and 2 µg/mL concentrations. This is coupled with misshapen or irregular cells, primarily in the higher 20 µg/mL and 200 µg/mL concentrations, which made it difficult to distinguish individual cells. In contrast, untreated cells maintained regular cell shape, consistent cell sizing and defined cell borders.

**Fig. 3:**
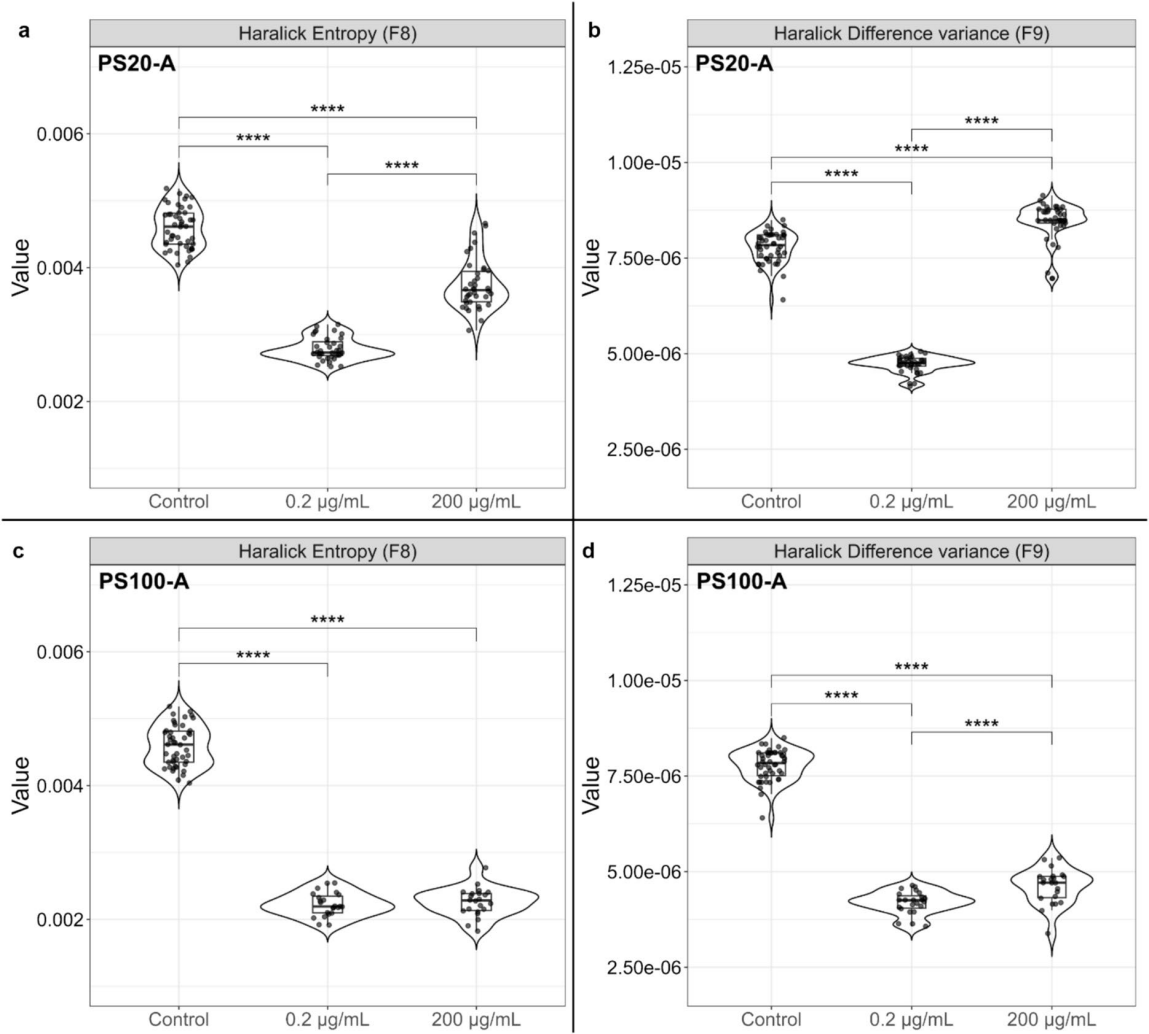
Assessment of cell morphology via haralick entropy and difference of variance in HK-2 cells following 24-h of NPs exposure. Cell morphology was assessed via QuPath cell segmentation and analysis comparing 0.2 µg/mL and 200 µg/mL NPs concentrations. **a**) Entropy and **b**) Difference of Variance of PS20-A NPs. **c**) Entropy and **d**) Difference of Variance of PS100-A NPs. N ≥ 22 ROI. Data are representative of individual replicates tested and normalised to the ROI area. Adjusted P-value =  **** ≤ 0.0001

A cell segmentation analysis was performed to quantify changes to cell granularity and morphology following NPs exposure (Fig. S8). When compared to the control group, the cells exposed to NPs treatments systematically expressed a significant reduction (Adjusted P-value < 0.0001) in the mean pixel intensity (Fig. S9). While there appears to be no visual morphological differences across all concentrations of the PS20-A NPs (Fig. 2 and S6), increases in the exposure concentration from 0.2 µg/mL to 200 µg/mL was associated with significant increases in the entropy and heterogeneity of the cells, 34.4% and 78.8% respectively (Fig. 3a and b). In contrast, the PS100-A treatment group displayed noticeable granulation within the cytoplasm of cells exposed to the two highest NPs concentrations, localised around the nuclei of the cells (Fig. 2 and S6), associated with a 10.2% increase in cell heterogeneity (Fig. 3d). While the source of the granulation is unclear, this could be a result of aggregation and accumulation of the PS NPs within the cytoplasm. This granulation is also visible and significantly increased in the 200 µg/mL PE50-B and PMMA50-B NPs treatment groups (Adjusted P-value < 0.0001) (Fig. 2, Fig. S10). Both sizes of PMMA NPs from manufacturer C aggregated so significantly at 200 µg/mL that the cells became difficult to visualise, thus no real difference between the two sizes could be identified (Fig. 2). This aggregation also impacted the cell segmentation analysis and were excluded from the granularity analysis as a result.

### Live/dead analysis

**Fig. 4:**
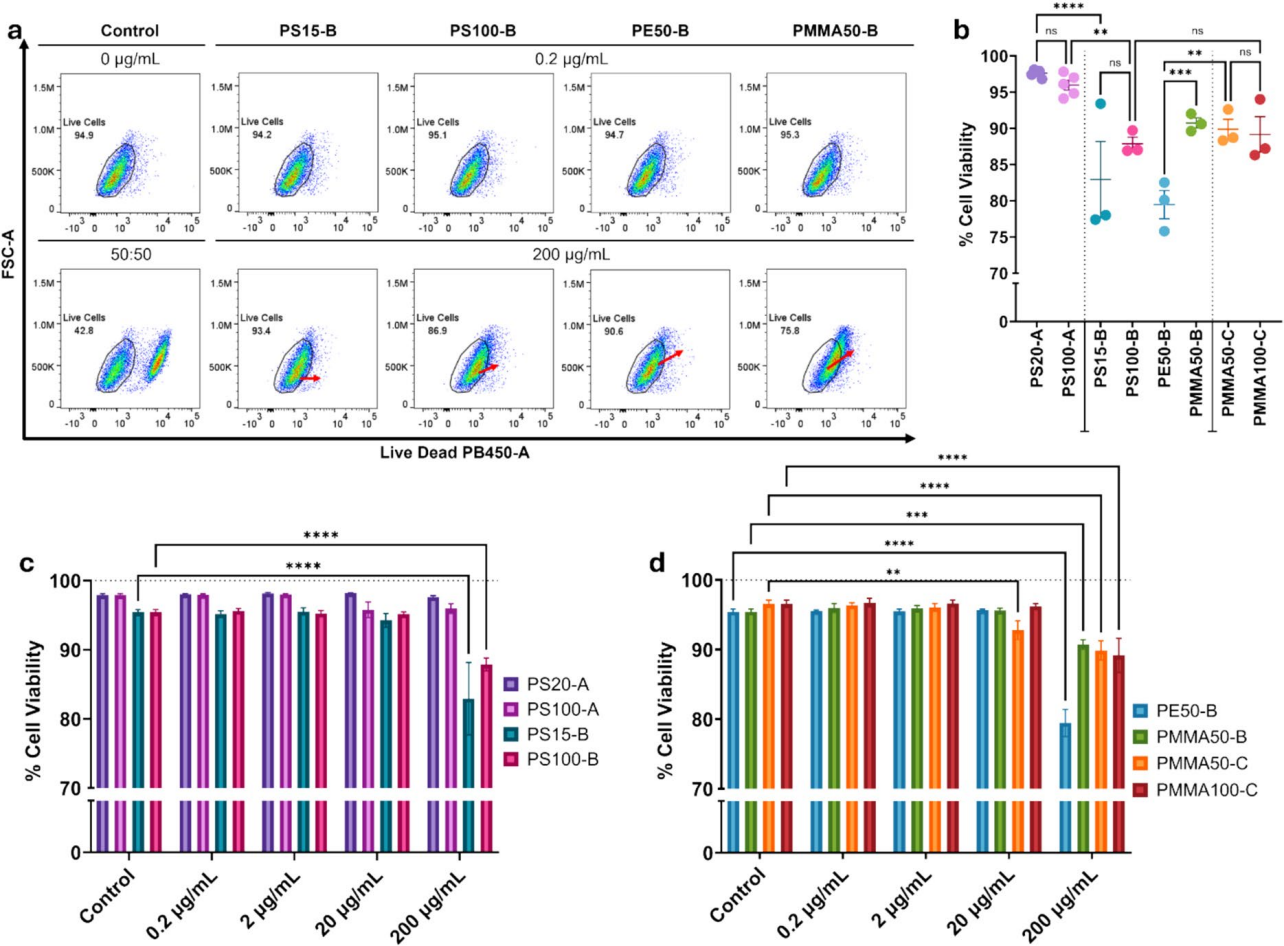
Cell viability of HK-2 cells exposed to various sizes and concentrations of PS, PMMA and PE NPs for 24-h. Viability was assessed via flow cytometry using Live/Dead Aqua to fluorescently label dead cells. Cells were treated with increasing concentrations from 0.2 µg/mL to 200 µg/mL. **a**) Flow cytometry assessment of viability comparing NPs from Manufacturer B at 0.2 µg/mL and 200 µg/mL. Red arrows indicate the directional shift of the population. **b**) Comparison of cell viability after exposure 200 µg/mL concentrations of various NPs polymers, sizes and manufacturers. **c**) Comparison of cell viability after exposure to PS NPs from Manufacturer A and B. **d**) Comparison of cell viability after exposure to PE and PMMA NPs from Manufacturer B and C. *N* = 3. In the whisker and bar plots (b-d), data are representative of replicate experiments and is expressed as mean with standard error of the mean. *P*-value = ‘ns’ no significance, ** ≤ 0.01, *** ≤ 0.001, **** ≤ 0.0001

Cell viability was assessed by live/dead flow cytometry assay. In the flow cytometry plots, the live cell population shifts away from the live cell gate, towards the dead cell zone as the concentration of NPs increases from 0.2 µg/mL to 200 µg/mL (Fig. 4a, S11 and S12). Despite these noticeable shifts in the scatterplots, statistical significance was typically seen only at the highest NPs concentration. Specifically, after 24-h exposure to the PS20-A and PS100-A NPs, there was no significant reduction in cell viability when comparing concentrations. This is in direct contrast to the PS15-B and PS100-B NPs which, following exposure at 200 µg/mL, resulted in a significant reduction in cell viability of 12.5% (P-value < 0.0001) and 7.6% (P-value < 0.0001) respectively, when compared to the control (Fig. 4b and c).

This concentration dependent population shift can also be seen in the PE50-B and PMMA50-B treatment groups (Fig. 4a). Cell viability was significantly reduced by 16% (P-value < 0.0001) and 4.7% (P-value 0.0008), after exposure to 200 µg/mL of the respective PE50-B and PMMA50-B NPs (Fig. 4d). When comparing the two sizes and manufacturers of PMMA, the reduced viability was similar between the three treatment groups. The difference in viability between the PMMA50-B and PMMA50-C NPs is 0.87% and non-significant. Similarly, the difference in viability between the two sizes (50 and 100 nm) from Manufacturer C was 0.7% and non-significant. This result agrees with their measured DLS sizes and illustrates that nominal size labels do not always correspond to distinct NPs populations. Interestingly, across all treatment groups, the largest reduction in cell viability was seen after treatment with the PE50-B NPs at a concentration 200 µg/mL, reducing viability to 79.46% (P-value < 0.0001). This could imply a link between the type of polymer and cell viability.

An additional experiment was completed with both sizes of PS NPs from Manufacturer A in which the NPs were spiked with NaN_3_ and Tween-20 to determine if the presence of these additives in NPs solutions was contributing to the cell death seen at the higher concentrations. After exposure to the spiked NPs for 24-h, the cells treated with spiked PS100-A NPs saw increased cell death at 20 µg/mL and 200 µg/mL (Fig. S13). The positive controls of cells treated with NaN_3_ and Tween-20 without NPs showed no change to cell viability on their own.

### Cell cycle analysis

**Fig. 5:**
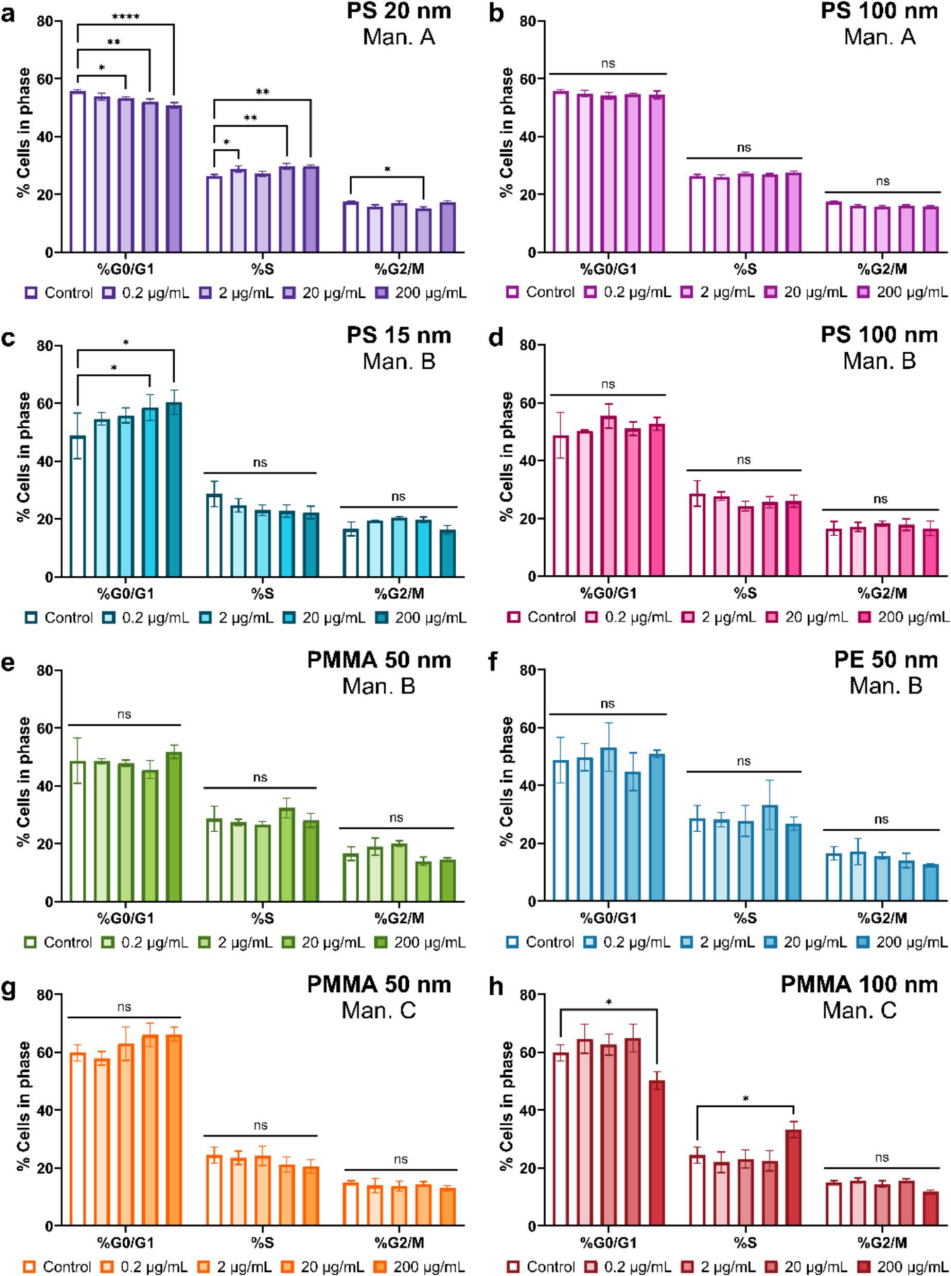
Exposure of HK-2 cells to varying sizes and concentrations of NPs resulted in dysregulation of the cell cycle. Cells were treated for 24-h with NPs at concentrations from 0.2 µg/mL to 200 µg/mL. Cell cycle was assessed via flow cytometry utilising propidium iodide (PI) staining. **a**) Proportion of cells in each phase after treatment with PS20-A NPs. **b**) Proportion of cells in each phase after treatment with PS100-A NPs. **c**) Proportion of cells in each phase after treatment with PS15-B NPs. **d**) Proportion of cells in each phase after treatment with PS100-B NPs. **e**) Proportion of cells in each phase after treatment with PMMA50-B NPs. **f**) Proportion of cells in each phase after treatment with PE50-B NPs. **g**) Proportion of cells in each phase after treatment with PMMA50-C NPs. h) Proportion of cells in each phase after treatment with PMMA100-C NPs. Data are representative of replicate experiments and is expressed as standard error of the mean. N = 3. *P*-value = ‘ns’ no significance, * ≤ 0.05, ** ≤ 0.01, **** ≤ 0.0001

Following exposure to varying size and concentrations of PS, PE and PMMA NPs, a cell cycle assay was performed to determine the proportion of cells in three distinct cell cycle phases, namely the G0/G1, S and G2/M phase (Fig. S14 and S15). Significant cell cycle dysregulation was seen in the cells treated with the smallest PS15-B and PS20-A NPs (Fig. 5a and c), with no significant changes observed in the larger PS100-A and PS100-B NPs from either manufacturer (Fig. 5b and d). Cells treated with the PS15-B NPs exhibited a dose-dependent increase in the percentage of cells in the G0/G1 phase (5.8% to 11.6%), with a significant increase in the 20 µg/mL and 200 µg/mL treatment groups (Fig. 5c). In the PS20-A NPs treatment group, the opposite was seen, with significant decreases of the percentage of cells in the G0/G1 phase observed in a dose-dependent manner (Fig. 5a). A corresponding shift was seen in the percentage of cells in the S phase (2.4% to 3.4%), with significant increases in the cells of the 0.2, 20 and 200 µg/mL treatment groups, and significant decreases in the percentage of cells in the G2/M phase of the 20 µg/mL group (Fig. 5a).

Interestingly, when assessing the effect of NPs of other polymer types, the opposite is seen when compared to PS NPs. No significant effect to the cell cycle was seen in the cells exposed to the PE50-B, PMMA50-B and PMMA50-C NPs (Fig. 5e-g), rather the cells exposed to the PMMA100-C NPs exhibited significant arrest at the S phase, with significant reduction in the percentage of cells in the G0/G1 phase at a concentration of 200 µg/mL (Fig. 5h).

### Nanoplastic internalisation

**Fig. 6:**
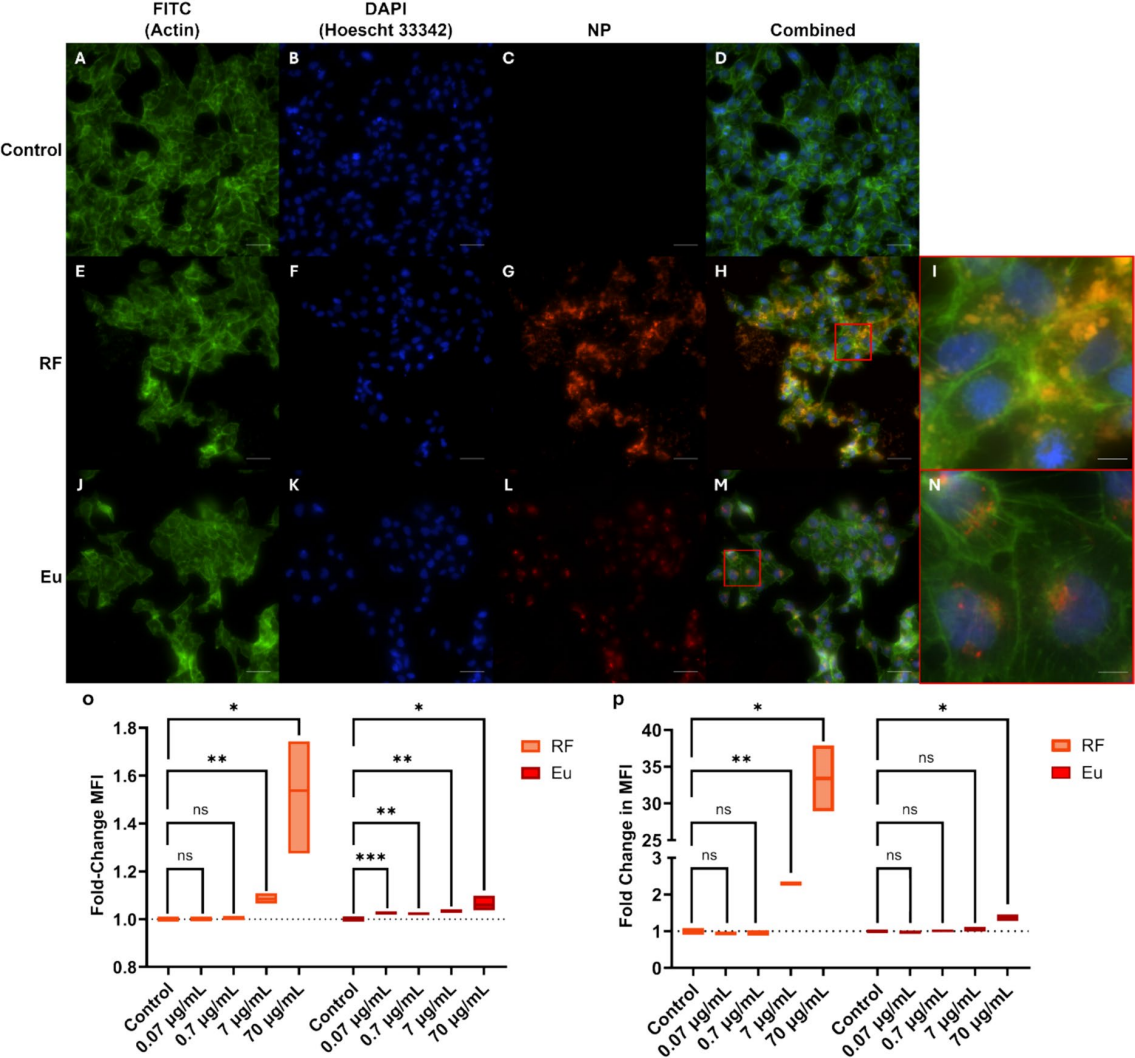
Internalisation of RF and Eu 100 nm PS NPs after 24-h of exposure. **a**-**n**) Fluorescence microscopy images of HK-2 cells following NPs exposure at 70 µg/mL. Cytoskeleton stained with Actin Green (Phalloidin), Nucleus stained with NucBlue (Hoechst 33,342). Scale bar = 50 µm. Scale bar on zoomed images = 10 µm. **o**) MFI of red fluorescent (RF) and Europium-labelled (Eu) 100 nm PS NPs at 0.07 to 70 µg/mL (1.36 × 10^8^ to 1.36 × 10.^11^ particles/mL). **p**) Flow cytometry assessment of RF and Eu NPs internalisation. Data are representative of technical replicates. N = 3. *P*-value = ‘ns’ no significance, * ≤ 0.05, ** ≤ 0.01, *** ≤ 0.001

The NPs internalisation study was initially conducted using two different 100 nm PS NPs to assess for potential fluorophore leaching: externally labelled RF PS NPs, and internally labelled Eu PS NPs. In both cases, the fluorescent NPs accumulated within the cytoplasm of the cell and often close to the nucleus (Fig. 6). Uptake of fluorescent NPs were only visible via microscopy at the highest NPs concentrations investigated, namely 7 µg/mL (Fig. S16) and 70 µg/mL concentrations (Fig. 6). After the 24-h exposure period, the cells exposed to the RF PS NPs typically displayed brighter aggregates, with apparent accumulation outside of the cell membrane. In contrast, cells exposed to the Eu PS NPs shone more dimly, but with clear accumulation around the nucleus (Fig. 6i and n).

Analysis of the mean fluorescence of the microscopy images indicated uptake of both the RF and Eu PS NPs (Fig. 6o). A significant increase was identified in the MFI of cells treated with concentrations of 7 µg/mL and 70 µg/mL of RF PS NPs, suggesting a dose dependent uptake of NPs. In comparison, all concentrations of Eu PS NPs expressed significant MFI increases when compared to the control group, with the largest proportion of uptake seen in the 70 µg/mL treatment group (Fig. 6o). Internalisation of both fluorescent PS NPs was also assessed via flow cytometry as an alternate way to assess NPs uptake (Fig. 6p). Much like the microscopy images, the MFI as expressed from the flow cytometry data indicated significant increases in NPs uptake in the 7 µg/mL (*P*-value 0.0003) and 70 µg/mL (*P*-value 0.0093) concentrations of the RF PS NPs. In the Eu PS NPs treatment group, the MFI of the 70 µg/mL concentration was significantly increased (P-value 0.0263) when compared to the 7 µg/mL concentration (Fig. 6p).

Taken together, these results indicate uptake of both RF- and Eu-labelled PS NPs, with concentration-dependent increases observed for each fluorophore. In neither case was fluorescence detected in the exposure media, indicating that any potential dye loss was not substantial enough to mask uptake. RF-NPs— commercially available in the required range of polymers and sizes—could therefore be used without expecting dye leakage to confound the interpretation of polymer- and size-dependent effects.

**Fig. 7:**
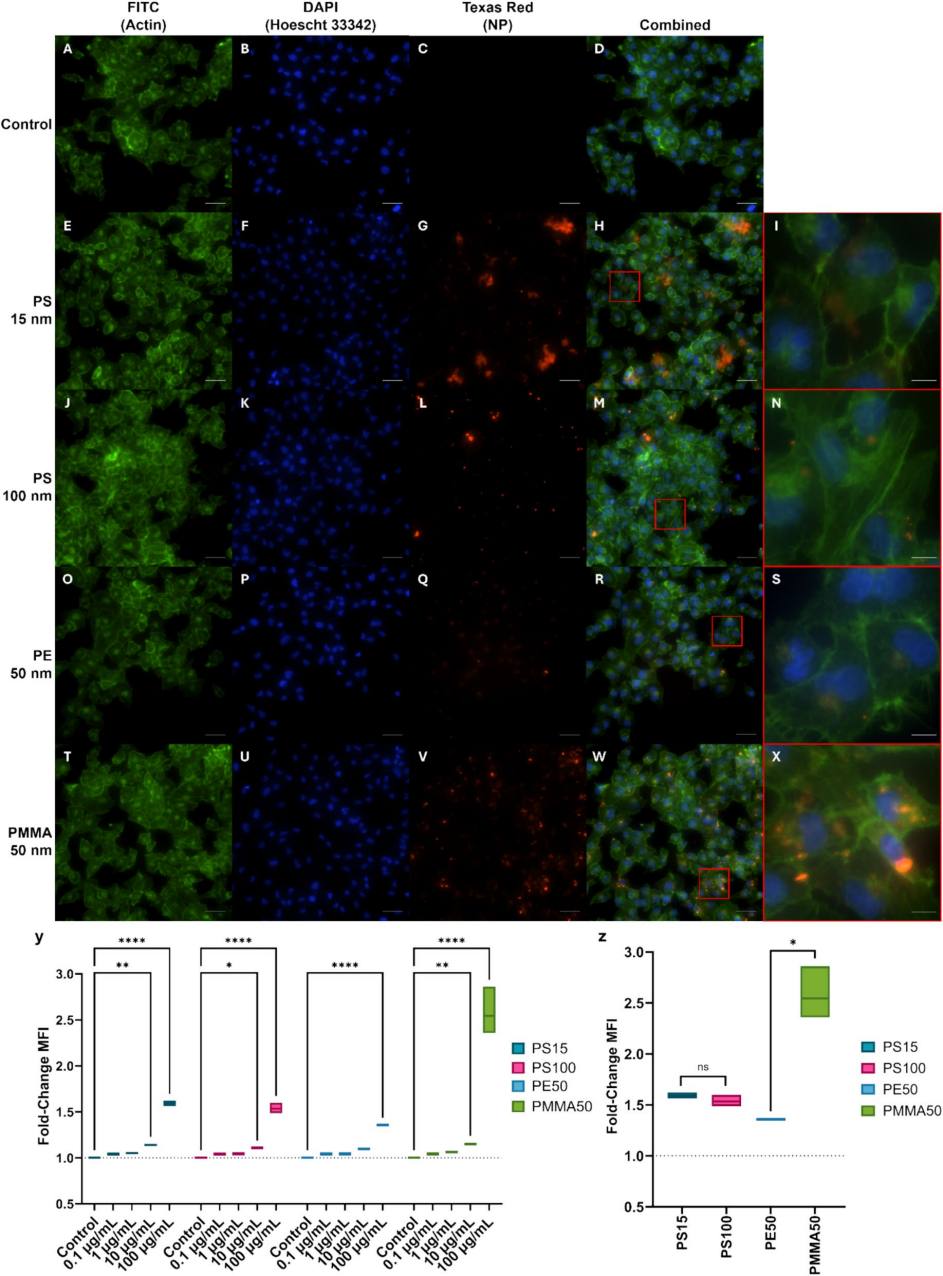
Internalisation of RF NPs after 24-h of exposure. 100 µg/mL. **A**–**X**) Fluorescence microscopy images of HK-2 cells following NPs exposure. Cytoskeleton stained with Actin Green (Phalloidin). Nucleus stained with NucBlue (Hoechst 33342). Scale bar = 50 µm. Scale bar on zoomed images = 10 µm. Y) MFI of fluorescent PS, PE and PMMA NPs at concentrations of 0.1, 1, 10 and 100 µg/mL. Z) MFI of fluorescent PS, PE and PMMA NPs at 100 µg/mL. Data are representative of technical replicates. *N* = 3. *P*-value = ‘ns’ no significance, * ≤ 0.05, ** ≤ 0.01, **** ≤ 0.0001

Further analysis of NPs internalisation was performed to assess for differences in uptake between different polymers and sizes of NPs (Fig. 7). To evaluate the effect of NPs size, two populations of PS NPs were used, PS15-B-RF and PS100-B-RF. When visualising the cells treated with PS15-B-RF NPs, internalisation of the NPs within the cytoplasm was not visible at this magnification, although significant particle aggregation was seen to accumulate on the exterior of the cell (Fig. 7h and i). The cells exposed to the PS100-B-RF NPs presented similarly to the initial internalisation experiment, with brighter aggregates of the particles that appear to sit outside of the cell membrane (Fig. 7m and n). To evaluate the effect of polymer type, two populations of 50 nm NPs were used, PE50-B-RF and PMMA50-B-RF. The PMMA50-B-RF treatment group showed some similarities to the PS100-B-RF group, presenting a mix of bright aggregates on the outer membrane of the cell, with dimmer NPs populations that appear to be accumulated within the cell membrane (Fig. 7w and x). Most interestingly, the cells exposed to the PE50-B-RF NPs almost exclusively express dimmer NPs populations that had been internalised within the cell membrane. Within these populations, a clear point of accumulation can be seen within each cell, which often overlaps immediately adjacent to the cell’s nucleus (Fig. 7r and s). This localisation was further confirmed via confocal microscopy (Fig. S17). The difference in uptake between the different NPs polymer types, particularly with the PE NPs, may indicate that some polymers are able to be more readily internalised by cells.

Analysis of the MFI from the microscopy images also indicates a dose-dependent uptake of the NPs within a 24-h exposure period (Fig. 7y). Increased MFIs are seen at all concentrations for all NPs treatment groups, with a significant increase in the 100 µg/mL groups. The largest increase in MFI was seen in the cells treated with PMMA50-B-RF, which is likely to be attributed to the combination of internalised NPs and aggregates adhered to the outer cell membrane, noting that the relative fluorescence intensity is not a direct measure of particle number. With the PE50-B-RF treated cells having the lowest increase in MFI at 100 µg/mL, which could be attributed to the strict internalisation of the particles (Fig. 7z).

## Discussion

Over recent years, there has been growing concern about the potential harm that degraded plastics pose to human health. MPs in particular have been shown to affect several physiological systems, including the kidneys (Liang et al. 2021; Meng et al. 2022). NPs, however, represent a newer and less understood area of research, especially regarding the influence of particle size and polymer type. In this study, we investigated the effects of NPs of varying polymer types, sizes, and concentrations on HK-2 cells to evaluate differences in cell viability, internalisation, and cell-cycle alterations.

### Effect of concentration

Although there is a growing number of articles investigating the effects of NPs, there are still considerable variations in the concentrations used and the resultant effects on cells, for example, on viability (Jeong et al. 2022; Kihara et al. 2021; Sarma et al. 2022). Concentration-dependent effects of 80 nm PS NPs on the human renal tubular epithelial cell line HKC were demonstrated in a study by Wang et al. (2022), where a significant reduction in cell viability was associated with NPs concentrations of 80 µg/mL, 100 µg/mL, and 300 µg/mL. This study also found that spectrofluorometric analysis showed that increases in concentration correlated with increased NPs internalisation (Wang et al. 2022).

In our in vitro studies, we chose the human cell line, HK-2 cells, which were generated from proximal tubules of the kidney. Under our conditions, we found that HK-2 cells were largely unaffected when exposed to NPs of less than 100 µg/mL, though significant changes were most prominent at higher concentrations (100 µg/mL and 200 µg/mL), which included morphological changes and loss of viability. Similarly, Chen et al. (2025) also demonstrated loss of viability from 200 µg/mL when exposing HK-2 cells to NPs over a 24-h period. According to their studies, viability only decreased by roughly 10% at the highest concentration of 800 µg/mL (Chen et al. 2025). Given that physiological concentrations of NPs are unlikely to reach such levels in the kidney, we selected 200 µg/mL as our maximal concentration, which was also shown by Chen et al. (2025) to increase intracellular ROS production.

### Effect of polymer

Previous studies have shown that surface functionalisation influences NPs toxicity, with carboxylic acid (-COOH) or amine (-NH_2_) modified PS NPs generally found to be more harmful than pristine particles, and -COOH less toxic than -NH_2_ modification (Chen et al. 2023; Gonzalez-Fernandez et al. 2021; Wang et al. 2023). Here all NPs were functionalised with -COOH, and all had negative surface charge (Fig. 1q-x), yet polymer-dependent differences persisted, suggesting that the chemical composition of the polymer itself may also be a determinant of NPs toxicity, not just surface charge. Our data, for example, showed that PE50-B NPs (Fig. 4c)had the most pronounced effect on viability, whilst the PMMA100-C NPs also promoted S-phase arrest at 200 µg/mL (Fig. 5h). The PS100-B NPs on the other hand, despite having a similar change in viability and equivalent NaN_3_ concentration as the PMMA100-C NPs, did not induce cell cycle alterations.

The potential for variation in internalisation and toxicological impact between NPs of different polymers could be largely explained by the differences in composition, structure and characteristics of the particles. Polymeric properties such as surface energy, polar regions, and molecular structure all play a role in a particle’s ability to adsorb substances onto its surface, leading to the formation of protein coronas, which, in turn, can influence biological interactions (Kihara et al. 2021; Shi et al. 2024). For instance, when directly comparing the effect of 100 nm PS, PE and polypropylene (PP) NPs on the normal human lung epithelial cell line, BEAS-2B, Dou et al. (2026) noted polymer-associated differences after 24-h of exposure. More than 50% cell death was observed across all NPs, with dose-dependent decreases in viability at 100 µg/mL and 200 µg/mL of PE and PP NPs, while the PS NPs were the most toxic, with significant impact to the cell viability at 50 µg/mL and near complete cell death at the two highest concentrations (Dou et al. 2026). Differential polymer-dependent effects on viability and cell cycle arrest seen in this and our study, suggest that environmental mixtures of NPs may exert heterogeneous effects, underscoring the need to assess multiple polymer types in toxicology studies.

### Effect of size

The impact of NPs size has been shown in a study performed by Li et al. (2023), in which PS NPs 20 nm, 60 nm and 100 nm in size, comparable to those used in our study, were exposed to human embryonic kidney cells, where it was seen that the 20 nm PS NPs were significantly more toxic than the larger NPs, expressing near complete cell death at the 75 µg/mL and 125 µg/mL concentrations tested, compared to ~ 80% viability for the cells exposed to the 60 nm and 100 nm PS NPs (Li et al. 2023). Other studies assessing exposure to PS NPs above 100 nm in size, including MPs, consistently showed no impact on kidney cell viability, regardless of concentration or exposure time (Goodman et al. 2022; Li et al. 2023; Wang et al. 2022). In our study, we found that HK-2 cell viability responses remained largely unchanged across all NPs sizes at all concentrations below 200 µg/mL. These differences could be attributed to the use of human embryonic cells used by Li et al. (2023). The HEK293 cell line, derived from human embryonic kidney tissue, lacks a tissue specific gene expression signature, and instead exhibits neuronal-like characteristics and likely originated from adrenal gland tissue (Stepanenko and Dmitrenko 2015). A recent study comparing the gene expression profiles of renal proximal tubule epithelial cells found that HEK293 cells had the least similarity to the human kidney medulla and were among the lowest performing cell lines for nephrotoxicity predictability (Sakolish et al. 2025).

Despite lack of significant viability impact, we did find cell cycle alterations with the two smallest sizes of PS (15 nm and 20 nm) causing dysregulation after 24-h of exposure, even at concentrations as low as 0.2 µg/mL (PS20-A NPs, Fig. 5a and b). This was the only case in which such a low concentration produced a statistically significant impact, suggesting that smaller sizes of PS NPs are more readily able to interfere with cell growth and proliferation. The mechanism of increased S-phase arrest cannot be discerned from these studies alone. As S-phase arrest is commonly associated with DNA damage (Chao et al. 2017), this is one potential mechanism, and subsequent studies of DNA damage could be undertaken to explore this. These effects were observed in azide-free particles (Manufacturer A), ruling out attribution to this toxic additive. Taken together, the cell cycle findings may be indicative that smaller NPs sizes cause DNA damage, warranting investigation in longer-term exposure studies.

The only size-related difference observed in the internalisation assay conducted here was the size of the NPs aggregates, with the small NPs only visible as larger, more diffuse, aggregates, likely due to resolution limits. It is worth noting here, that single 15 or 20 nm NPs or small aggregates of them would not be detectable via microscopy.

### Additives and aggregation

The presence of additives, or lack thereof, within NPs suspensions also appeared to impact viability and cell response, particularly for PS NPs from Manufacturer B which contained NaN_3_ and Tween-20. These NPs significantly reduced HK-2 cell viability compared to additive-free PS-NPs from manufacturer A, where viability was largely unchanged (Fig. 4c).

When PS NPs from Manufacturer A were spiked with NaN_3_ and Tween-20, cell viability decreased, despite the additives on their own showing no effects at equivalent concentrations (Fig. S13). This result could infer some level of synergistic biological effect where exposure to the additives makes the cells more susceptible to NPs-induced toxicity. Unfortunately, often the impact of surfactants added to commercial NPs suspension cannot be assessed due to non-disclosure of the exact formulation by the manufacturer. Yet, commonly used surfactants, such as Triton X-100, are known to have dose-dependent toxic effects, which may correlate with dose-dependent toxicity seen in NPs studies (Vercauteren et al. 2025). Given the known cytotoxicity of NaN_3_ even at low concentrations (Heinlaan et al. 2020; Petersen et al. 2022; Pikuda et al. 2019), and the potential for surfactants to also contribute toxic effects, NPs studies should aim to include controls that specifically gauge the effect of these additives. The findings presented here in Fig. S13 illustrate that undisclosed additives in commercial NPs suspensions can meaningfully alter cellular responses, reinforcing the need for cautious interpretation. Whether the observed effects of additives result from membrane degradation, altered permeability, or increased endocytic/phagocytic activity within the cell, is yet to be determined.

In addition to additives, aggregation of NPs is another factor that could impact biological responses. In this study we saw PS15-B, PMMA50-C and PMMA100-C NPs had a tendency to aggregate within the treatment period, which was confirmed by DLS analysis of the NPs in media (Fig. S4), and may have an impact on the cell’s response (Fig. 2, S6 and S7).

### Outlook and future work

This study used an immortalised proximal tubule cell line, commercially produced spherical NPs, and a short 24-h exposure period, which cannot fully replicate the complexity of human kidney tissue or long-term exposure scenarios. Primary kidney cells or 3-dimensional spheroid models could provide additional physiological relevance in future works. Commercial NPs also differ from environmentally derived particles, which are typically more heterogeneous in shape, size and composition. This is a significant and common limitation across NPs research into cellular and systemic effects and toxicity. The NPs used in this study were of a consistent size (Fig. 1), sphericity and composed of a single polymer type, and while not mimicking environmentally relevant scenarios, the controlled particle properties used here allowed us to isolate and directly compare the effects of polymer type, size, and concentration on kidney cell responses. Future research would benefit from investigating NPs that have been aged to better mimic the physicochemical properties of environmental NPs, alongside multi-polymer mixes, and common environmental contaminants to obtain more nuanced understanding of current environmental exposure. Further work is also needed to define the mechanisms behind NPs internalisation and toxicity. Experiments assessing uptake would aid in determining not only the mode of uptake, but also the localisation of NPs within cells, and lead to further understanding of how NPs exposure can impact cellular functioning. Analysis of renal or kidney-specific biomarkers would also help elucidate mechanistic effects of NPs exposure.

While the lower concentrations tested in this study may be indicative of short-term environmental exposure, real-world contact with NPs is more likely to occur at even lower concentrations than the ones tested in this study, but over an extended period of time. For instance, a recent study by Ten Hietbrink et al. (2025) evaluated concentrations of NPs smaller than 1 µm within the North Atlantic Ocean, identifying an average NPs concentration of 15.1 mg/m^3^ (0.015 µg/mL equivalent), with PS equating to 4.06 mg/m^3^ (0.004 µg/mL equivalent). These concentrations are only 1 order of magnitude smaller than the lowest concentration tested here. The potential cumulative effects and delayed biological impacts cannot be reflected in short-term, acute exposure models. However, a recent study by Nihart et al. (2025) saw a 2.3 µg per gram increase of plastic in kidney tissue between 2016 and 2024, which seems to indicate that bioaccumulation does occur, and may do so in a more pronounced fashion as environmental exposure increases. Hence the concentrations used here may become more and more biologically relevant as plastic pollution increases over time. For now, short-term studies like this one are instead useful for assessing the immediate impacts, cellular responses and toxicity of specific NPs types, and offer a framework for designing longer term experiments with environmentally relevant exposure thresholds necessary to further understanding of possible toxicity, effect and health impacts.

## Conclusion

The findings of the study demonstrate that while lower concentrations of NPs may not result in immediate toxicity to the HK-2 cell line, particularly in terms of short-term exposure, higher NPs burdens can compromise overall cell health and function, affecting morphology, viability and cell cycle regulation. The results also indicate that NPs effects are influenced not only by concentration but also by polymer composition and particle size, with some combinations inducing significant cellular changes even at relatively low doses. Given that proximal tubule cells play a critical role in reabsorption and overall kidney filtration efficiency, sustained or repeated damage to these cells could impair kidney function, potentially leading to NPs accumulation in kidney tissue, reduced clearance capacity, and increased NPs recirculation in the bloodstream over time. This highlights the importance of investigating more realistic, environmentally relevant NPs exposure, that reflect chronic, low-level contact over extended periods, and that account for the diversity of NPs characteristics in terms of concentration, size, polymer types and chemical additives likely to be encountered in real-world settings. Such studies should also explore mechanistic endpoints, including potential DNA damage and long-term functional consequences, to fully assess the risks posed by environmental NPs to kidney health and systemic exposure.

## Supplementary Information

Below is the link to the electronic supplementary material.

Supplementary file1 (DOCX 11.4 MB)

## Data Availability

No datasets were generated or analysed during the current study.
