# Supplementary material for: Nanoplastic toxicity and uptake in kidney cells: differential effects of concentration, particle size, and polymer type
Source: Cell Biol Toxicol. 2026 Jan 16;42(1):21. doi: 10.1007/s10565-025-10135-2 (PMC12835115; doi:10.1007/s10565-025-10135-2)
Supplement: Supplementary file 1 — Supplementary file1 (DOCX 11.4 MB) [file 10565_2025_10135_MOESM1_ESM.docx]

# Nanoplastic toxicity and uptake in kidney cells: differential effects of concentration, particle size, and polymer type

Authors:

Hayden Louis Gillings,*^a, b, c^ Darling M Rojas-Canales,^b, d^ Soon Wei Wong,^b,^ ^d^ Kaustubh R. Bhuskute,^e, f^ Amandeep Kaur,^e, f^ Iliana Delcheva,^a^ Jonathan M Gleadle,^b, d^ Melanie MacGregor *^a, c^

Affiliations:

^a^ College of Science and Engineering, Flinders University, Sturt Road, Bedford Park, South Australia, 5042, Australia

^b^ Flinders Health and Medical Research Institute, College of Medicine and Public Health, Flinders University, Sturt Road, Bedford Park, South Australia, 5042, Australia

^c^ Nano and Microplastics Research Consortium, Flinders University, Sturt Road, Bedford Park, South Australia, 5042, Australia

^d^ Department of Renal Medicine, Flinders Medical Centre, Flinders Drive, Bedford Park, South Australia, 5042, Australia

^e^ Medicinal Chemistry, Monash Institute of Pharmaceutical Sciences, Monash University, Parkville, Melbourne, Victoria 3052, Australia

^f^ Australian Research Council Centre of Excellence for Innovations in Peptide and Protein Science, Monash University, Melbourne, Victoria 3052, Australia

* Corresponding Authors

[hayden.gillings@flinders.edu.au](mailto:hayden.gillings@flinders.edu.au)

[melanie.macgregor@flinders.edu.au](mailto:melanie.macgregor@flinders.edu.au)

# Supplementary Information

### Nanoplastic Particle Details and Calculations

Table S1 Summary of NP treatment concentrations and particle numbers across NP exposure experiments. All NPs were diluted in PBS and cell culture media to a final ratio of 1:10 PBS to media. Particles/mL was calculated using Equation 1. Particles per cell was calculated using Equation 2. *As provided by manufacturers

| Supplier | Polymer | Manufacturer Label* (nm) | Manufacturer Reported Diameter* (nm) | Cat No. | Lot No. | Additives? | Density (g/cm^3^) | Concentration (g/mL) | Concentration (µg/mL) | Particles/mL | Well Volume (µL) | Number of Cells | Particles per Cell |
| --- | --- | --- | --- | --- | --- | --- | --- | --- | --- | --- | --- | --- | --- |
| Thermo Fisher Scientific  (A) | PS | 20 | 28 | C37261 | 2418536 |  | 1.055 | 2×10^-4^ | 200 | 1.56×10^13^ | 500 | 0.1×10^6^ | 7.80×10^07^ |
|  |  |  |  |  |  |  |  | 2×10^-5^ | 20 | 1.56×10^12^ |  |  | 7.80×10^06^ |
|  |  |  |  |  |  |  |  | 2×10^-6^ | 2 | 1.56×10^11^ |  |  | 7.80×10^05^ |
|  |  |  |  |  |  |  |  | 2×10^-7^ | 0.2 | 1.56×10^10^ |  |  | 7.80×10^04^ |
|  |  | 100 | 110 | C37485 | 2646517 |  | 1.055 | 2×10^-4^ | 200 | 2.71×10^11^ | 500 | 0.1×10^6^ | 1.36×10^06^ |
|  |  |  |  |  |  |  |  | 2×10^-5^ | 20 | 2.71×10^10^ |  |  | 1.36×10^05^ |
|  |  |  |  |  |  |  |  | 2×10^-6^ | 2 | 2.71×10^09^ |  |  | 1.36×10^04^ |
|  |  |  |  |  |  |  |  | 2×10^-7^ | 0.2 | 2.71×10^08^ |  |  | 1.36×10^03^ |
| Lab261  (B) | PS | 15 | 12.5 | PST15C | 116437 | ✓ | 1.05 | 2×10^-4^ | 200 | 1.86×10^14^ | 500 | 0.1×10^6^ | 9.32×10^08^ |
|  |  |  |  |  |  |  |  | 2×10^-5^ | 20 | 1.86×10^13^ |  |  | 9.32×10^07^ |
|  |  |  |  |  |  |  |  | 2×10^-6^ | 2 | 1.86×10^12^ |  |  | 9.32×10^06^ |
|  |  |  |  |  |  |  |  | 2×10^-7^ | 0.2 | 1.86×10^11^ |  |  | 9.32×10^05^ |
|  |  | 100 | 89 | PST100C | 113973 | ✓ | 1.05 | 2×10^-4^ | 200 | 3.64×10^11^ | 500 | 0.1×10^6^ | 1.82×10^06^ |
|  |  |  |  |  |  |  |  | 2×10^-5^ | 20 | 3.64×10^10^ |  |  | 1.82×10^05^ |
|  |  |  |  |  |  |  |  | 2×10^-6^ | 2 | 3.64×10^09^ |  |  | 1.82×10^04^ |
|  |  |  |  |  |  |  |  | 2×10^-7^ | 0.2 | 3.64×10^08^ |  |  | 1.82×10^03^ |
|  | PMMA | 50 | 53 | PMMA50C | 215091 | ✓ | 1.18 | 2×10^-4^ | 200 | 2.17×10^12^ | 500 | 0.1×10^6^ | 1.09×10^07^ |
|  |  |  |  |  |  |  |  | 2×10^-5^ | 20 | 2.17×10^11^ |  |  | 1.09×10^06^ |
|  |  |  |  |  |  |  |  | 2×10^-6^ | 2 | 2.17×10^10^ |  |  | 1.09×10^05^ |
|  |  |  |  |  |  |  |  | 2×10^-7^ | 0.2 | 2.17×10^09^ |  |  | 1.09×10^04^ |
|  | PE | 50 | 68.2 | PE50C | 938761 | ✓ | <1 | 2×10^-4^ | 200 | 1.26×10^12^ | 500 | 0.1×10^6^ | 6.28×10^06^ |
|  |  |  |  |  |  |  |  | 2×10^-5^ | 20 | 1.26×10^11^ |  |  | 6.28×10^05^ |
|  |  |  |  |  |  |  |  | 2×10^-6^ | 2 | 1.26×10^10^ |  |  | 6.28×10^04^ |
|  |  |  |  |  |  |  |  | 2×10^-7^ | 0.2 | 1.26×10^09^ |  |  | 6.28×10^03^ |
| Phosphorex  (C) | PMMA | 50 | N/A | MMA50 | 44119 | ✓ | 1.19 | 2×10^-4^ | 200 | 2.58×10^12^ | 500 | 0.1×10^6^ | 1.29×10^07^ |
|  |  |  |  |  |  |  |  | 2×10^-5^ | 20 | 2.58×10^11^ |  |  | 1.29×10^06^ |
|  |  |  |  |  |  |  |  | 2×10^-6^ | 2 | 2.58×10^10^ |  |  | 1.29×10^05^ |
|  |  |  |  |  |  |  |  | 2×10^-7^ | 0.2 | 2.58×10^09^ |  |  | 1.29×10^04^ |
|  |  | 100 | N/A | MMA100 | 11062D | ✓ | 1.19 | 2×10^-4^ | 200 | 3.21×10^11^ | 500 | 0.1×10^6^ | 1.60×10^06^ |
|  |  |  |  |  |  |  |  | 2×10^-5^ | 20 | 3.21×10^10^ |  |  | 1.60×10^05^ |
|  |  |  |  |  |  |  |  | 2×10^-6^ | 2 | 3.21×10^09^ |  |  | 1.60×10^04^ |
|  |  |  |  |  |  |  |  | 2×10^-7^ | 0.2 | 3.21×10^08^ |  |  | 1.60×10^03^ |

Table S2 Summary of fluorescent NP treatment concentrations, volumes, and particle numbers for NP internalisation experiments. All NPs are diluted in PBS and cell culture media to a final ratio of 1:10 PBS to media. Particles/mL was calculated using Equation 1. Particles per cell was calculated using Equation 2. *As provided by manufacturers

| Supplier | Polymer | Manufacturer Label* (nm) | Manufacturer Reported Diameter* (nm) | Cat No. | Lot No. | Additives? | Fluorescent Label | Excitation (nm) | Emission (nm) | Density (g/cm^3^) | Concentration (g/mL) | Concentration (µg/mL) | Particles/mL | Well Volume (µL) | Number of Cells | Particles per Cell |
| --- | --- | --- | --- | --- | --- | --- | --- | --- | --- | --- | --- | --- | --- | --- | --- | --- |
| Magsphere | PS | 100 | 70-130 | CAFR100NM | CMFR4830 | ✓ | Red | 505-545 | 560-630 | 1.05 | 7×10^-5^ | 70 | 1.36×10^11^ | 100 | 0.1×10^5^ | 1.36×10^06^ |
|  |  |  |  |  |  |  |  |  |  |  | 7×10^-6^ | 7 | 1.36×10^10^ |  |  | 1.36×10^05^ |
|  |  |  |  |  |  |  |  |  |  |  | 7×10^-7^ | 0.7 | 1.36×10^09^ |  |  | 1.36×10^04^ |
|  |  |  |  |  |  |  |  |  |  |  | 7×10^-8^ | 0.07 | 1.36×10^08^ |  |  | 1.36×10^03^ |
| Thermo Fisher Scientific | PS | 100 | 96-98 | 93470350011150 | 270531 | ✓ | Europium (Internal) | 333 | 613 | 1.06 | 7×10^-5^ | 70 | 1.36×10^11^ | 100 | 0.1×10^5^ | 1.36×10^06^ |
|  |  |  |  |  |  |  |  |  |  |  | 7×10^-6^ | 7 | 1.36×10^10^ |  |  | 1.36×10^05^ |
|  |  |  |  |  |  |  |  |  |  |  | 7×10^-7^ | 0.7 | 1.36×10^09^ |  |  | 1.36×10^04^ |
|  |  |  |  |  |  |  |  |  |  |  | 7×10^-8^ | 0.07 | 1.36×10^08^ |  |  | 1.36×10^03^ |
| Lab261 | PS | 15 | 12.5 | FRP15C | 420359 | ✓ | Red | 545 | 566 | 1.05 | 1×10^-4^ | 100 | 9.32×10^13^ | 100 | 0.1×10^5^ | 9.32×10^08^ |
|  |  |  |  |  |  |  |  |  |  |  | 1×10^-5^ | 10 | 9.32×10^12^ |  |  | 9.32×10^07^ |
|  |  |  |  |  |  |  |  |  |  |  | 1×10^-6^ | 1 | 9.32×10^11^ |  |  | 9.32×10^06^ |
|  |  |  |  |  |  |  |  |  |  |  | 1×10^-7^ | 0.1 | 9.32×10^10^ |  |  | 9.32×10^05^ |
|  |  | 100 | 89 | FRP100C | 425092 | ✓ |  |  |  | 1.05 | 1×10^-4^ | 100 | 1.82×10^11^ | 100 | 0.1×10^5^ | 1.82×10^06^ |
|  |  |  |  |  |  |  |  |  |  |  | 1×10^-5^ | 10 | 1.82×10^10^ |  |  | 1.82×10^05^ |
|  |  |  |  |  |  |  |  |  |  |  | 1×10^-6^ | 1 | 1.82×10^09^ |  |  | 1.82×10^04^ |
|  |  |  |  |  |  |  |  |  |  |  | 1×10^-7^ | 0.1 | 1.82×10^08^ |  |  | 1.82×10^03^ |
|  | PMMA | 50 | 53 | FRPMMA50C | 624505 | ✓ |  |  |  | 1.18 | 1×10^-4^ | 100 | 1.09×10^12^ | 100 | 0.1×10^5^ | 1.09×10^07^ |
|  |  |  |  |  |  |  |  |  |  |  | 1×10^-5^ | 10 | 1.09×10^11^ |  |  | 1.09×10^06^ |
|  |  |  |  |  |  |  |  |  |  |  | 1×10^-6^ | 1 | 1.09×10^10^ |  |  | 1.09×10^05^ |
|  |  |  |  |  |  |  |  |  |  |  | 1×10^-7^ | 0.1 | 1.09×10^09^ |  |  | 1.09×10^04^ |
|  | PE | 50 | 89 | FRPE50C | 923012 | ✓ |  |  |  | 1.05 | 1×10^-4^ | 100 | 1.82×10^11^ | 100 | 0.1×10^5^ | 1.82×10^06^ |
|  |  |  |  |  |  |  |  |  |  |  | 1×10^-5^ | 10 | 1.82×10^10^ |  |  | 1.82×10^05^ |
|  |  |  |  |  |  |  |  |  |  |  | 1×10^-6^ | 1 | 1.82×10^09^ |  |  | 1.82×10^04^ |
|  |  |  |  |  |  |  |  |  |  |  | 1×10^-7^ | 0.1 | 1.82×10^08^ |  |  | 1.82×10^03^ |

Table S3 Input parameters for studied NPs systems and default settings used for RiskGONE In vitro dosimetry web application

| Nanoplastic particles parameters | | | |
| --- | --- | --- | --- |
| Material | PS | PMMA | PE |
| Density, g/cm^3^ | 1.05 | 1.19 | 0.97 |
| Effective density, g/cm^3^ | 1.05 | 1.19 | 0.97 |
| DLS distribution type | Fraction distribution by volume | | |
| Solvent parameters | | | |
| Density, g/cm^3^ | 0.9995 | | |
| Viscosity, P | 0.0081 | | |
| Temperature, °C | 37 | | |
| Simulation parameters | | | |
| Suspension column height, mm | 3 | | |
| Height of subcompartment, mm | 0.005 | | |
| Initial total concentration of material, mg/cm^3^ | 0.1 | | |
| Centrifugation (1 for gravity) | 1 | | |
| Total time of simulation, h | 24 | | |
| Time interval for simulation, s | 0.5 | | |
| Output parameters | | | |
| Time interval, min | 30 | | |
| Compartment height, mm | 0.01 | | |
| Output written for | Bottom area only | | |

### Supplementary Figures

FTIR-ATR spectra of the NPs was collected on a Thermo Fisher spectrometer Nicolet iS50 with a DuraScope ATR attachment with ZnSe crystal. A background scan was first collected. Following this, a 3.5 µL droplet of stock NPs solution was dropped on the ATR crystal, and was let to evaporate. The spectra of the polymer material, adsorbed on the crystal was then collected. Background and sample spectra were collected at 64 number of scans with 2 cm^-1^ resolution at spectral range 600 to 4000 cm^-1^.

Fig. S1 FTIR-ATR spectra of a) PS (library reference), b) PS20-A, c) PS15-B, and d) PS100-A

When comparing the reference PS spectrum (Fig. S1 a) with the spectra collected on PS20-A, PS100-A, PS15-B, and PS100-B, it is evident that the key peaks for PS are displayed.(Smith 2021b; Smith 1999) Among these are а prominent aromatic C-H bending out of plane peak at ~700 cm^-1^ and а smaller one at ~760 cm^-1^. Peaks associated with the aromatic C=C stretching at ~1490 cm^-1^ and 1600 cm^-1^ are also observed. Aromatic C-H in-plane bending is also displayed as expected at ~1025-1070 cm^-1^. The typical two types of C-H stretching for PS – aliphatic and aromatic – are also displayed at respectively, ~2850 and 2920 cm-1 and 3025 and 3080 cm-1. All of the tested PS NPs have a peak at 1452 cm^-1^ associated with CH_2_ bending.

Fig. S2 FTIR-ATR spectra of a) PMMA (library reference), b) PMMA50-B, c) PMMA50-C, and d) PMMA100-C

For all PMMA NPs studied, the characteristic strong ester carbonyl (C=O stretching) peak at about 1725 cm^-1^ is observed (Fig. S2).(Smith 2023; Smith 1999) The peaks associated with C-O-C stretching at ~1145-1190 cm^-1^ and ~1240-1260 cm^-1^ are also displayed. The symmetric and asymmetric peaks at ~2840-2990 cm^-1^ associated with CH_2_/CH_3_ stretching are observed. The C-H bending peak at ~1380 cm^-1^ is also displayed for all collected PMMA spectra.

Fig. S3 FTIR-ATR spectra of a) PE (library reference), and b) PE50-B

For the PE NPs sample, the typical symmetric and asymmetric CH_2_ stretches are observed at ~2848 cm^-1^ and ~2920 cm^-1^ (Fig. S3).(Smith 2021a; Smith 1999) The peak at ~1465 cm^-1^ associated with CH_2_ bending is also displayed. The sharp doublet at ~719 cm^-1^ and ~729 cm^-1^ suggests the NPs are HDPE.

Among the manufacturer B NPs, additional peaks such as polyether (C-O-C) stretching near the 1100-1130 cm^-1^ region are most evident in the PE spectrum. These are likely caused by the presence of Tween surfactant, used by manufacturer B.(Ortiz-Tafoya and Tecante 2018).

Fig. S4 Hydrodynamic diameter determined by DLS and plotted as a volume-weighted distribution for particles present in a) Cell culture media + 10% 1× PBS (control), as well as particles present in solutions of b) PS20-A, c) PS100-A, d) PMMA50-C, e) PMMA100-C, f) PS15-B, g) PS100-B, h) PMMA50-B, i) PE50-B dispersed in 1× PBS (top) and cell culture media + 10% 1× PBS (bottom). X-axis (hydrodynamic diameter) displayed on a base-10 logarithmic scale (log_10_). The particle populations observed around 10 nm (blue shading) are assigned to proteins. The multiple populations (red arrows) and/or broader peaks indicate partial aggregation


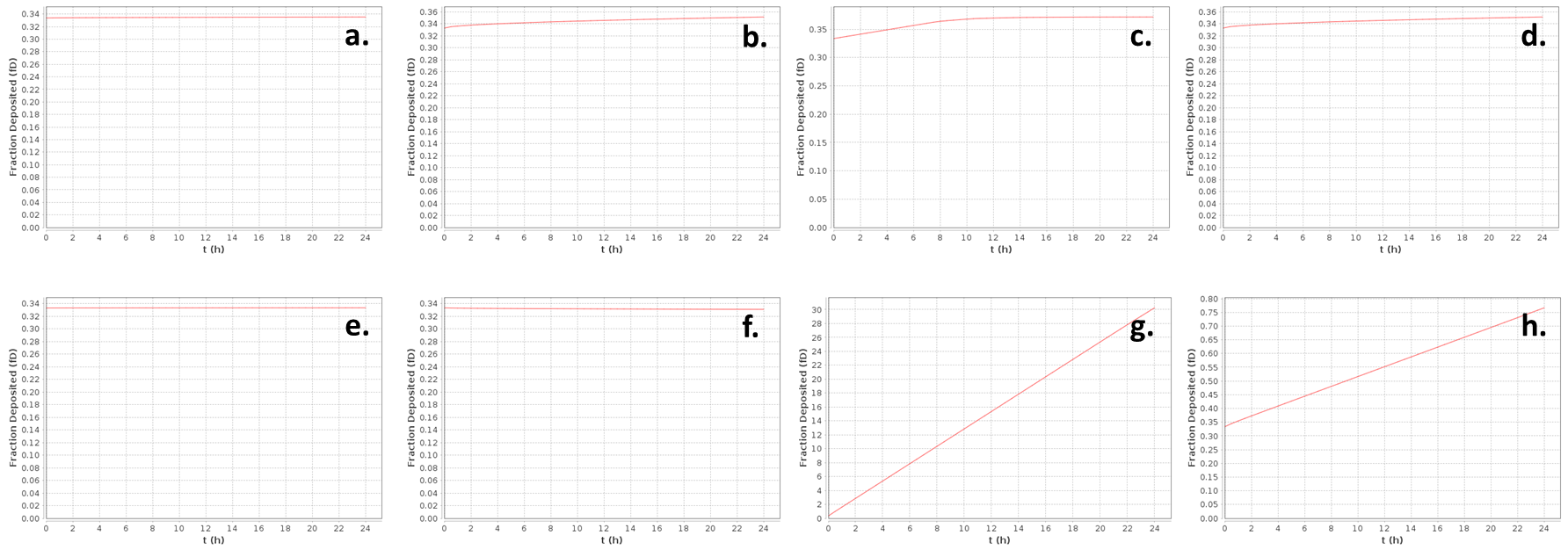


Fig. S5 Deposited fraction at the bottom-well over 24 h period simulated with RiskGONE In vitro dosimetry web application for a) PS20-A, b) PS100-A, c) PS15-B, d) PS100-B, e) PMMA50-B, f) PE50-B, g) PMMA50-C, and h) PMMA100-C. Input parameters are listed in Table S3


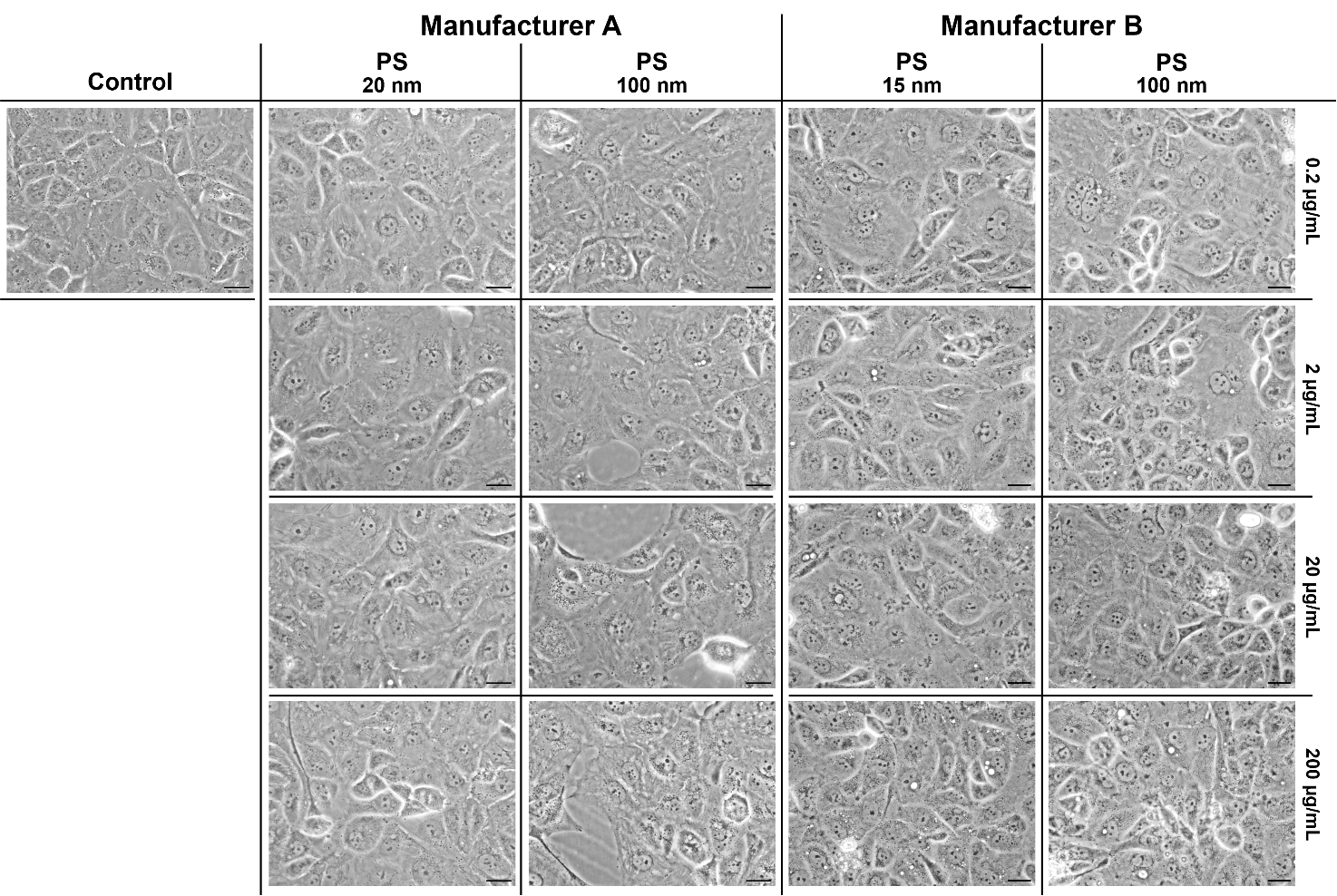


Fig. S6 Micrographs of HK-2 cells following 24-hour exposure to PS NPs of various sizes from two different manufacturers. All cells were treated with either PS20-A, PS100-A, PS15-B or PS100-B NPs at increasing concentrations from 0.2 µg/mL to 200 µg/mL, top to bottom. 40x magnification. Scale bar = 25 µm. N=3. Data is representative of individual replicates tested


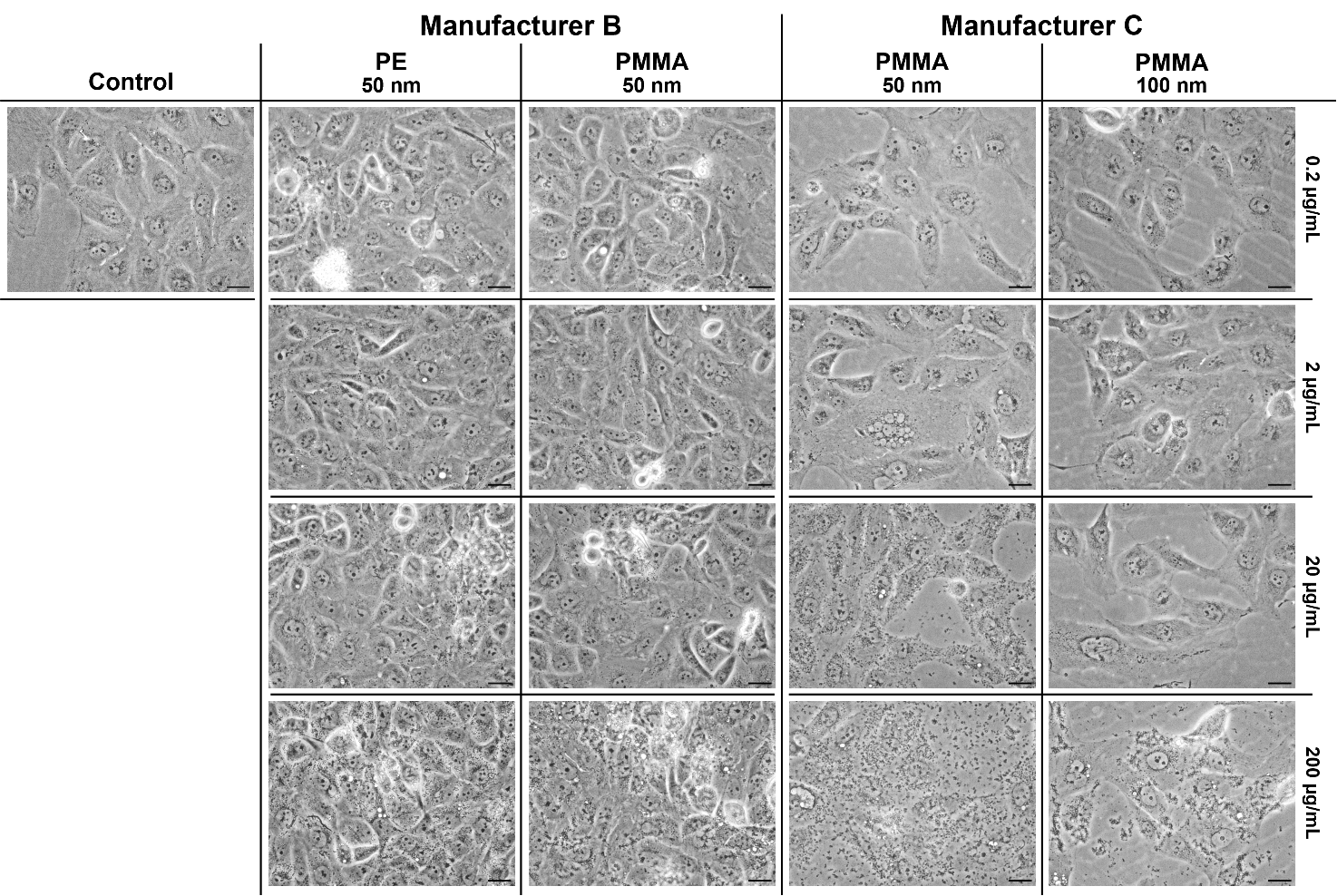


Fig. S7 Micrograph images of HK-2 cells exposed for 24-hours to various concentrations of NPs from multiple manufacturers. Cells were exposed to PE50-B, PMMA50-B, PMMA50-C and PMMA100-C NPs at increasing concentrations from 0.2 µg/mL to 200 µg/mL, top to bottom. 40x magnification. Scale bar = 25 µm. Data is representative of individual replicates tested. N=3


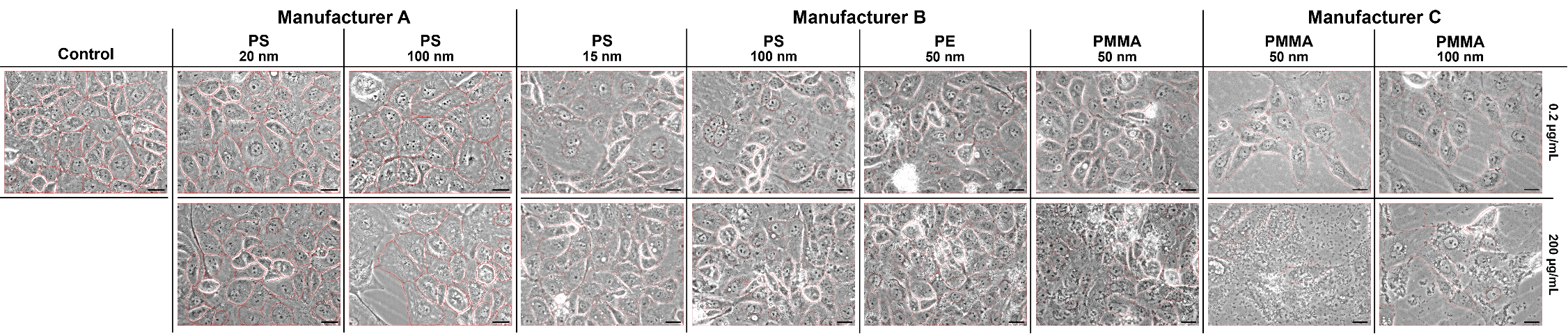


Fig. S8 Cell segmentation of HK-2 cell micrographs following 24-hour exposure to NPs of various sizes and polymers from three different manufacturers. All cells were treated with either PS20-A, PS100-A, PS15-B, PS100-B, PE50-B, PMMA50-B, PMMA50-C and PMMA100-C NPs at increasing concentrations from 0.2 µg/mL to 200 µg/mL. 40x magnification. Scale bar = 25 µm. Cell segmentation was performed using QuPath.


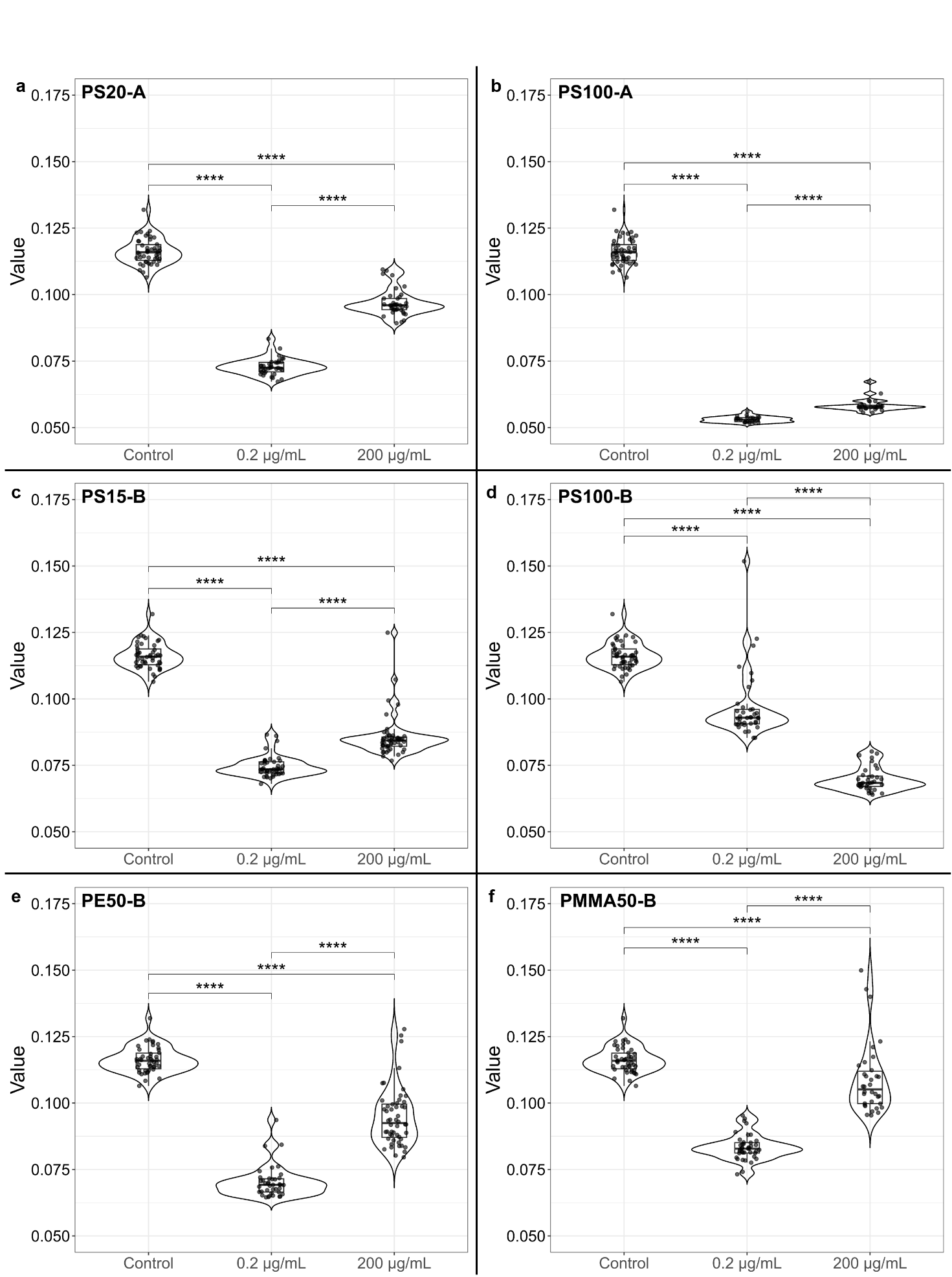


Fig. S9 Assessment of mean cell intensity in HK-2 cells following 24-hours of NP exposure. Cell morphology was assessed via QuPath cell segmentation and analysis comparing 0.2 µg/mL and 200 µg/mL concentrations of a) PS20-A, b) PS100-A c) PS15-B, d) PS100-B, e) PE50-B and f) PMMA50-B NPs. N ≥ 22 ROI. Data are representative of individual replicates tested and normalised to the ROI area. Adjusted P-value = **** ≤0.0001


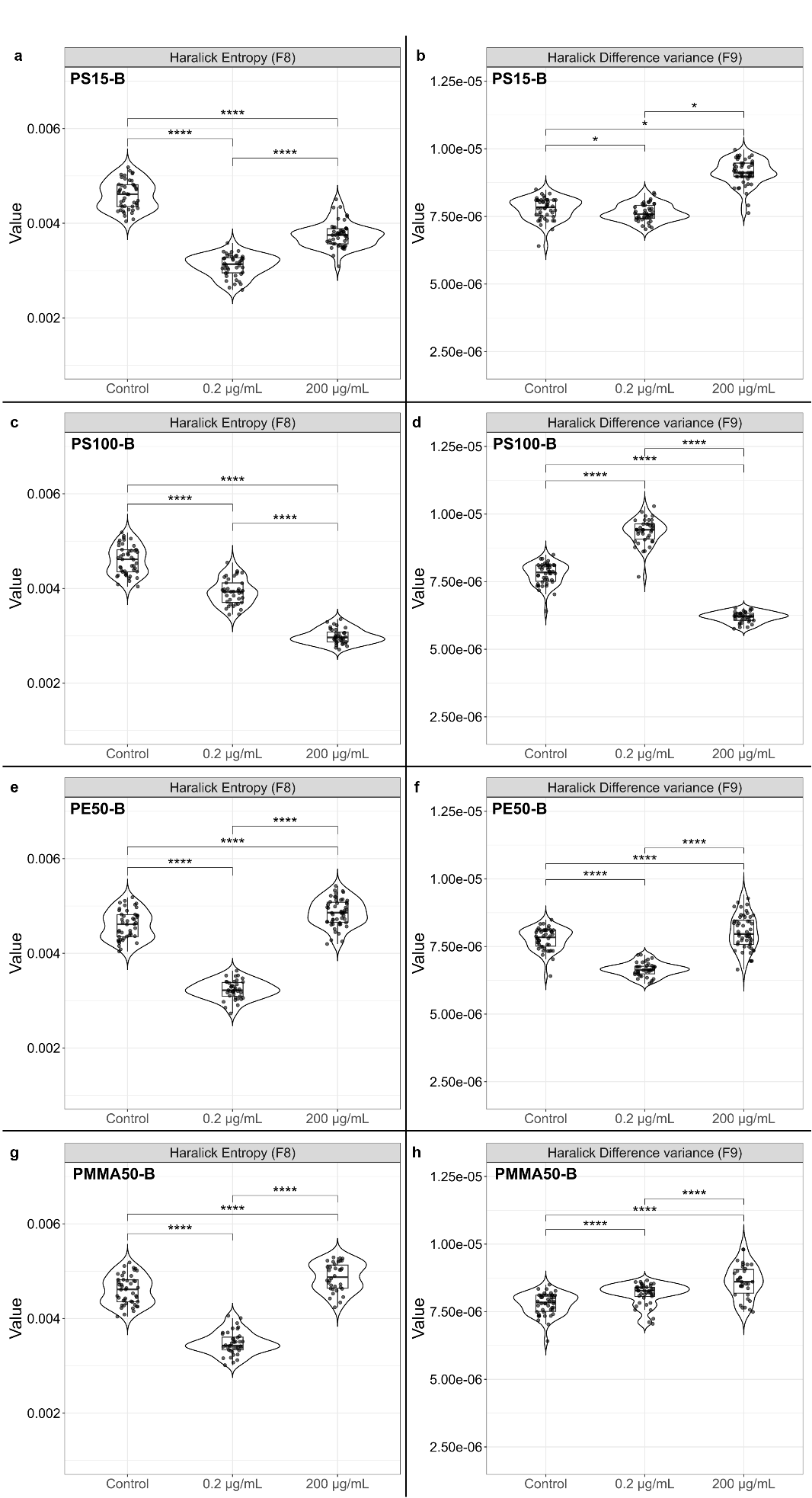


Fig. S10 Assessment of cell morphology via Haralick Entropy and Difference in Variance in HK-2 cells following 24-hours of NP exposure. Cell morphology was assessed via QuPath cell segmentation and analysis comparing 0.2 µg/mL and 200 µg/mL NP concentrations. a) Entropy and b) Difference of Variance of PS15-B NPs. c) Entropy and d) Difference of Variance of PS100-B NPs. e) Entropy and f) Difference of Variance of PE50-B NPs. g) Entropy and h) Difference of Variance of PMMA50-B NPs. N ≥ 22 ROI. Data is representative of individual replicates tested and normalised to the ROI area. Adjusted P-value = **** ≤0.0001


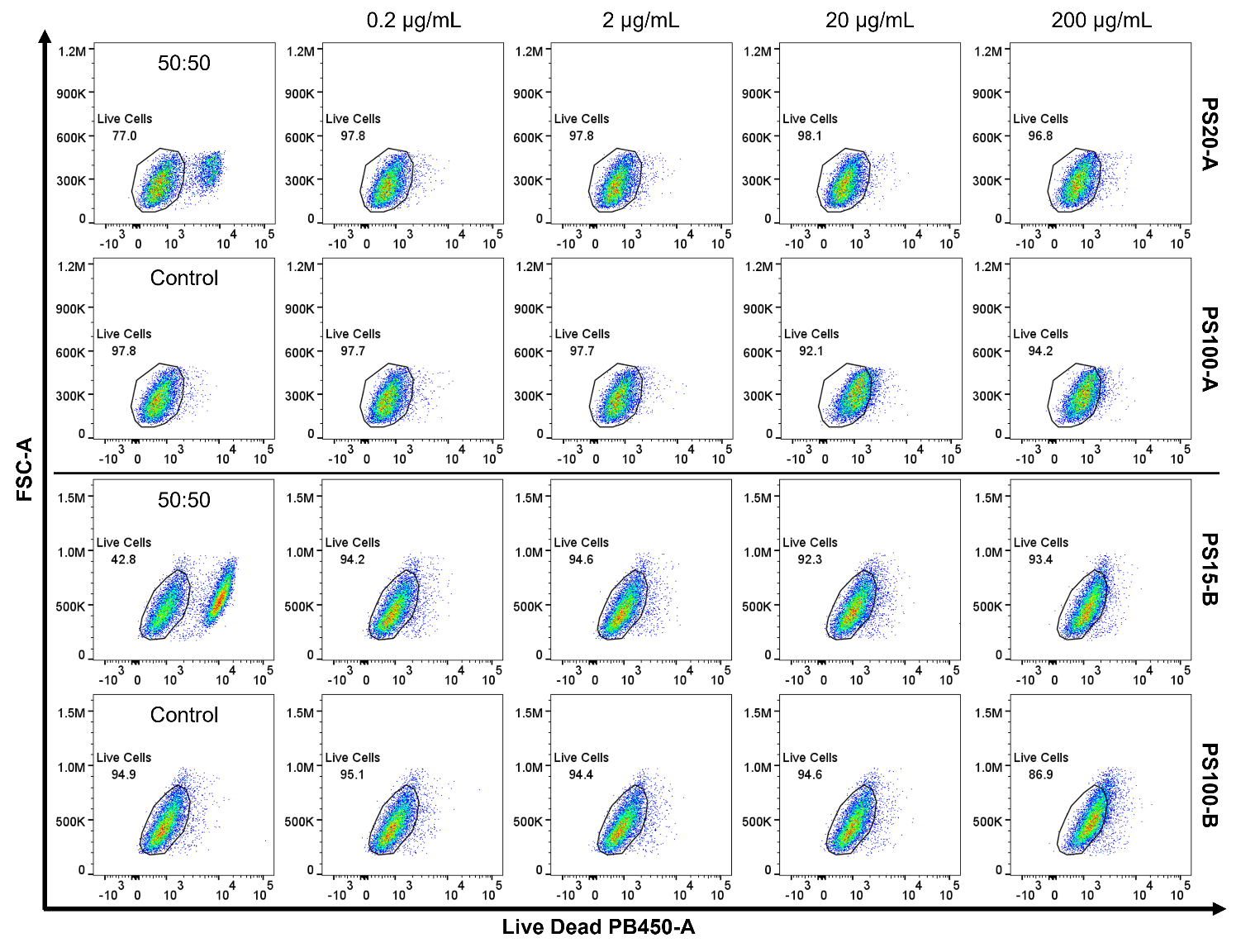


Fig. S11 Flow cytometry assessment of the viability of HK-2 cells after 24-hour exposure with varying concentrations and sizes of PS NPs from different manufacturers. Cell viability was assessed using LIVE/DEAD™ Fixable Aqua Dead Cell Stain. Cells were exposed with increasing concentrations of PS NPs from 0.2 µg/mL to 200 µg/mL (Left to right). N=3. Data is representative of individual replicates tested


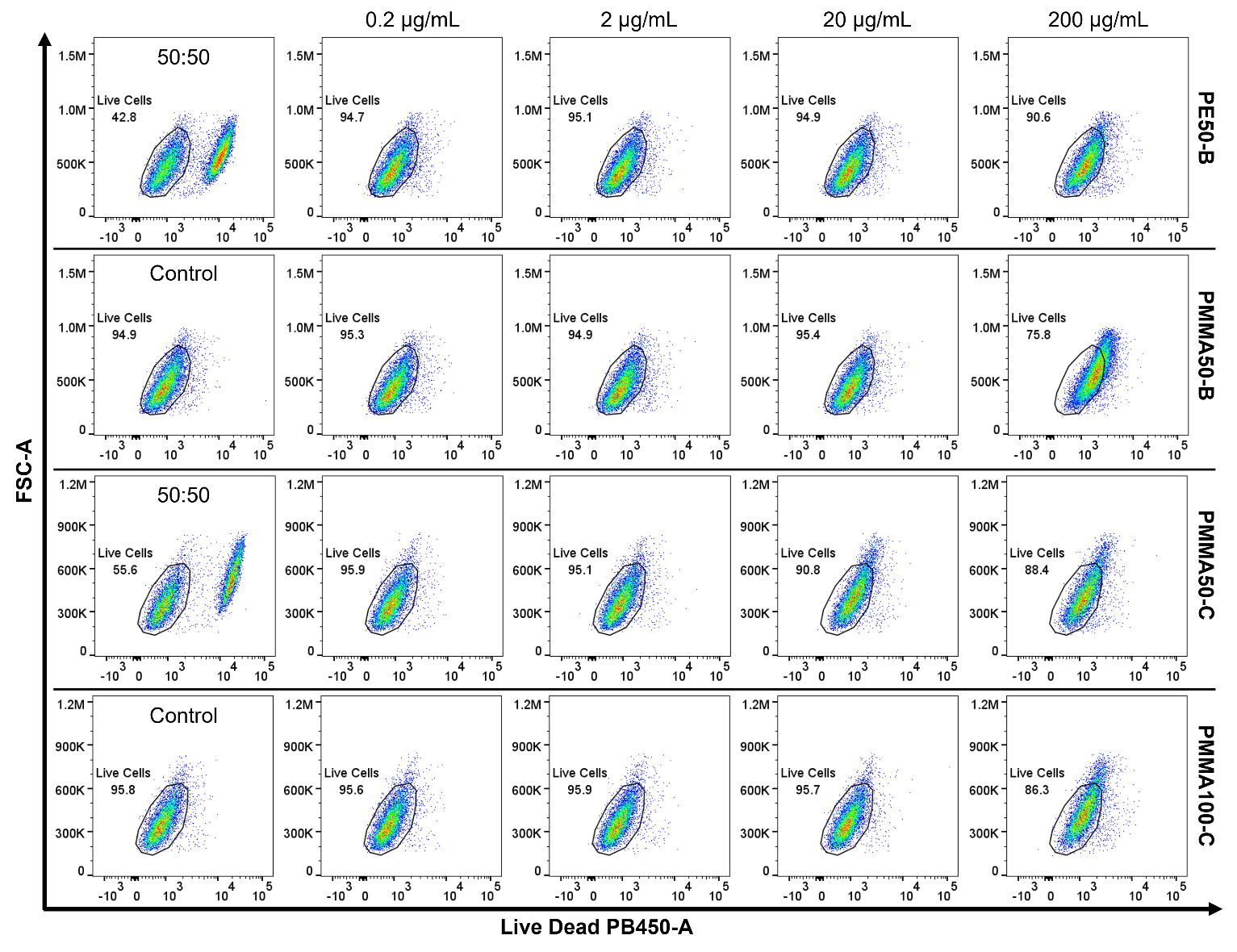


Fig. S12 Assessment of cell viability of HK-2 cells via flow cytometry after staining with LIVE/DEAD™ Fixable Aqua Dead Cell Stain. Cells were exposed to PE50-B, PMMA50-B, PMMA50-C and PMMA100-C NPs at concentrations of 0.2 µg/mL to 200 µg/mL (Left to right). N=3. Data is representative of individual replicates tested


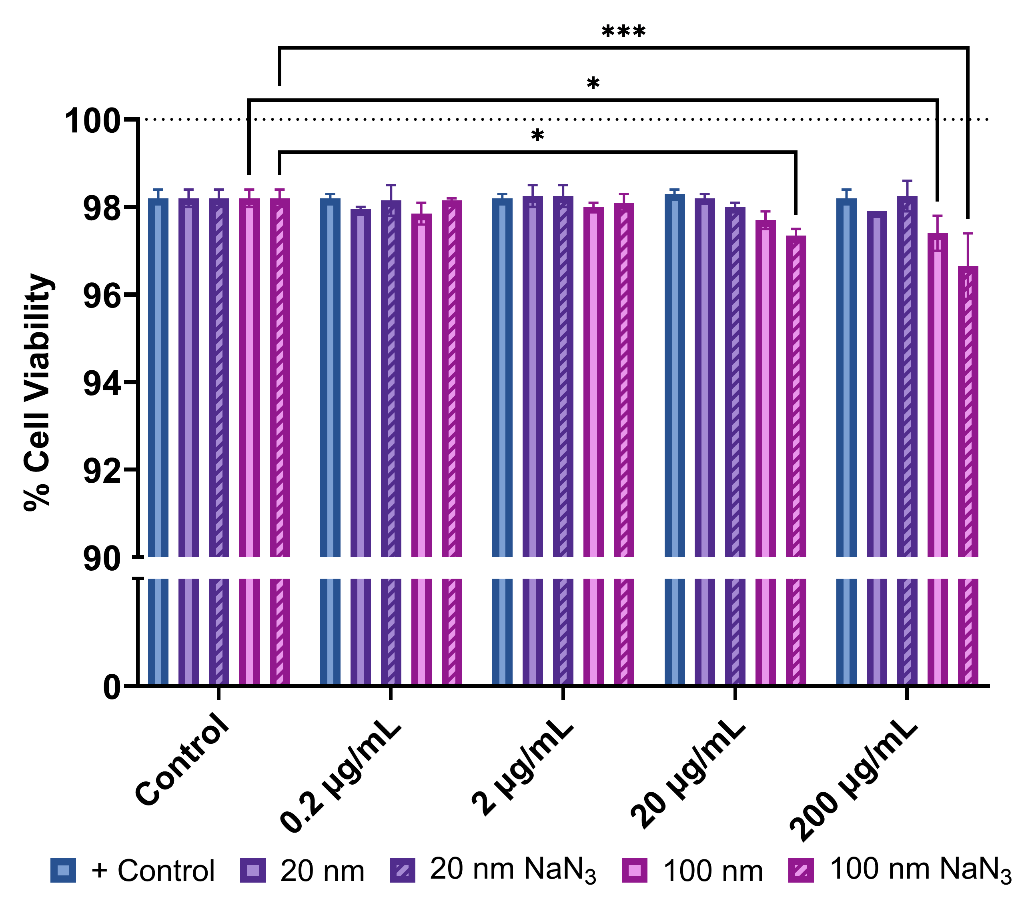


Fig. S13 Comparing the cell viability of HK-2 cells exposed to PS NPs spiked with NaN_3_ and Tween-20 after 24-hours of exposure at NP concentrations from 0.2 µg/mL to 200 µg/mL. Viability was assessed via flow cytometry using Live/Dead Aqua to fluorescently label the cells. Cells were treated with 20 nm and 100 nm PS NPs from Manufacturer A both as they were supplied and spiked with NaN_3_ and Tween-20 at equivalent concentrations to those supplied in NP suspensions from Manufacturer B. N=2. Data is representative of combined replicates and is expressed as mean with SEM. Significance was determined via two-way ANOVA with multiple comparisons Uncorrected Fisher’s least-significant difference test. P-value = *≤0.05, *** ≤0.001


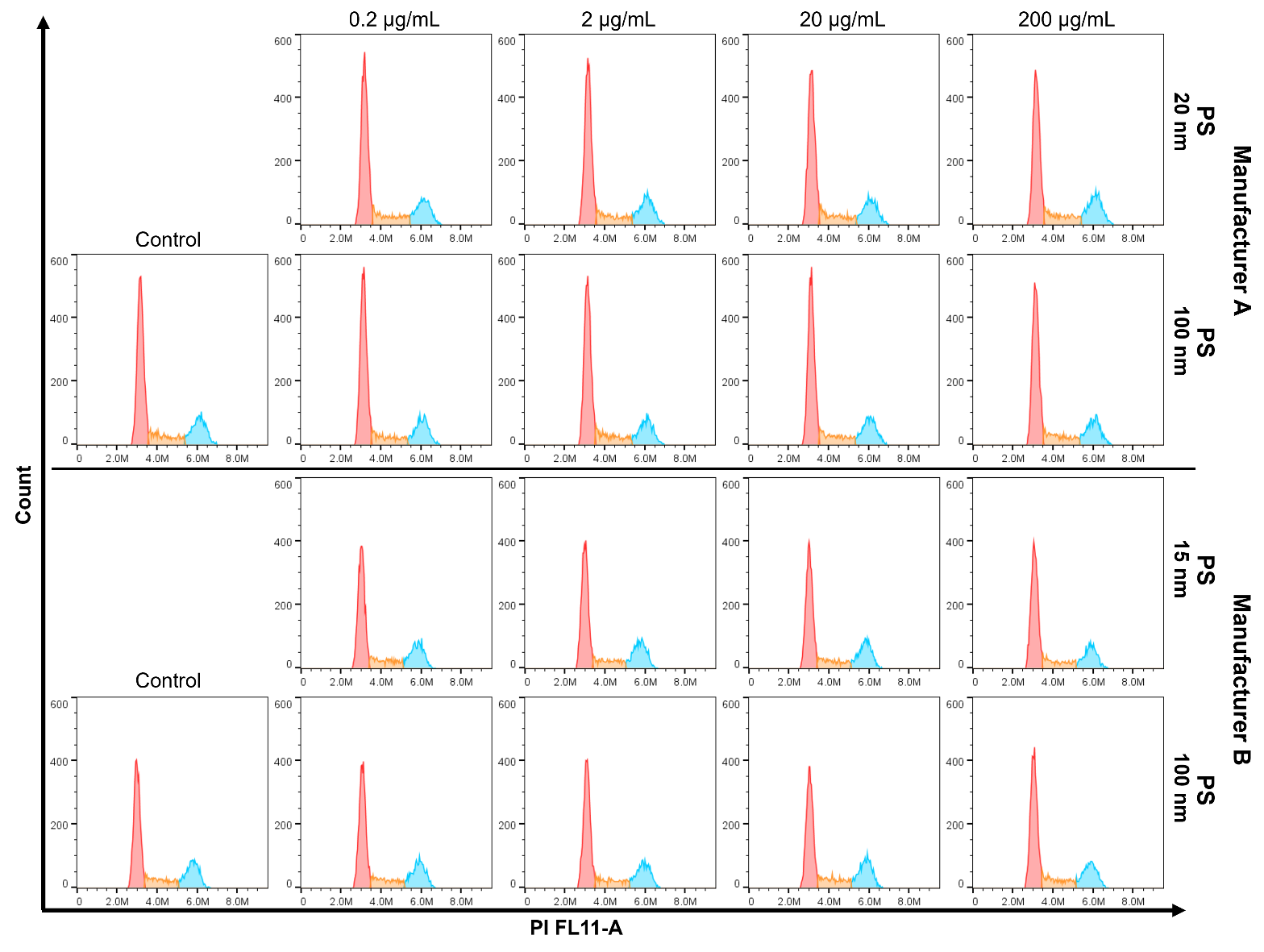


Fig. S14 Changes to cell cycle in HK-2 cells exposed to increasing concentrations of PS NPs. Cells were treated with concentrations of PS NPs from 0.2 µg/mL to 200 µg/mL for 24-hours, stained with PI and analysed via flow cytometry. G0/G1 phase = red peak, S phase = orange peak, G2/M phase = blue peak. Data is representative of individual replicates tested. N=3. Cell cycle phase distribution was modelled using the Watson Pragmatic algorithm within FlowJo (BD Biosciences)


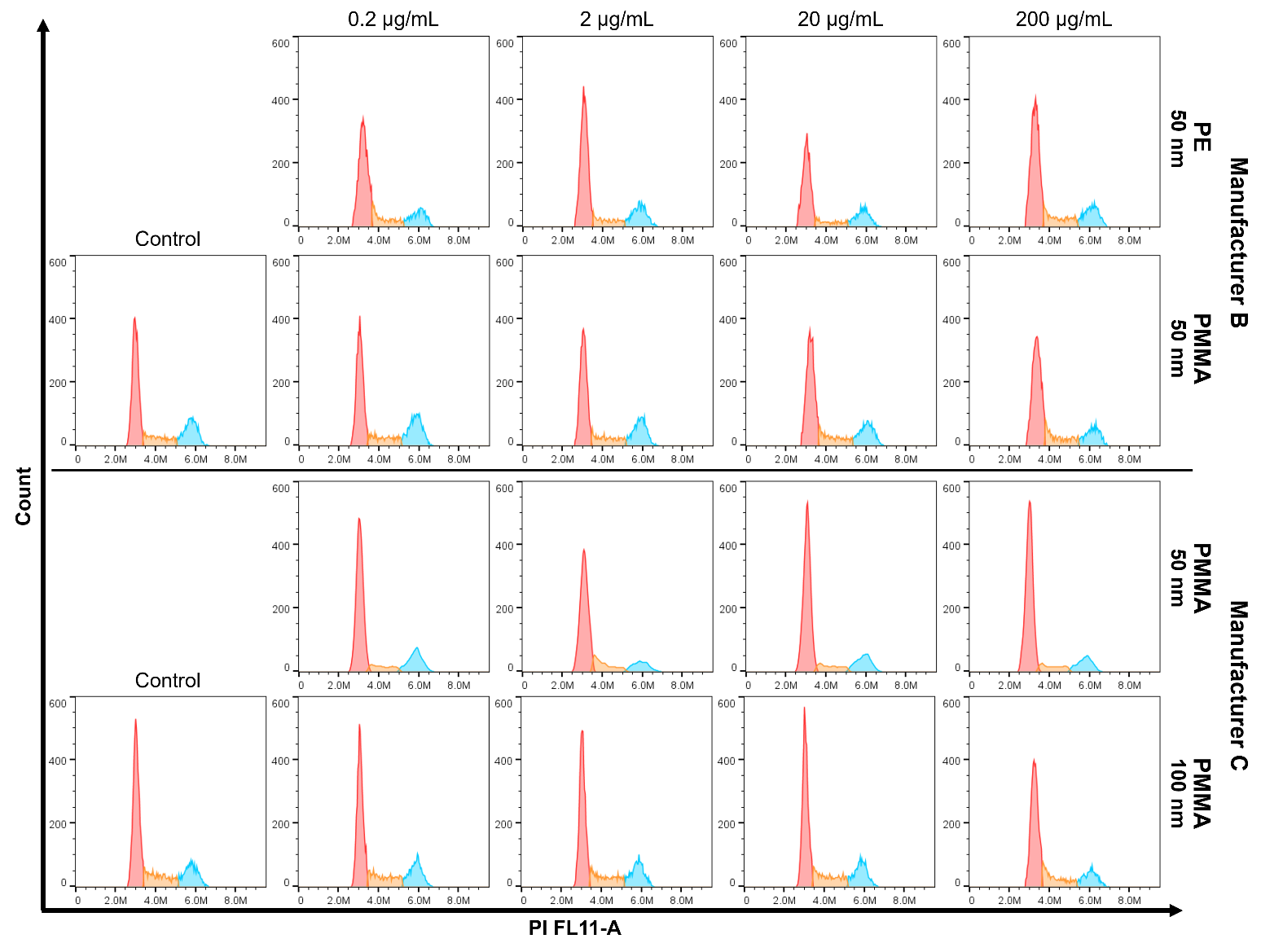


Fig. S15 Cell cycle changes in HK-2 cells exposed to PE and PMMA NPs at increasing concentrations. Cells were treated with concentrations of NPs from 0.2 µg/mL to 200 µg/mL for 24-hours, stained with PI and analysed via flow cytometry. G0/G1 phase = red peak, S phase = orange peak, G2/M phase = blue peak. Data is representative of individual replicates tested. N=3. Cell cycle phase distribution was modelled using the Watson Pragmatic algorithm within FlowJo (BD Biosciences)


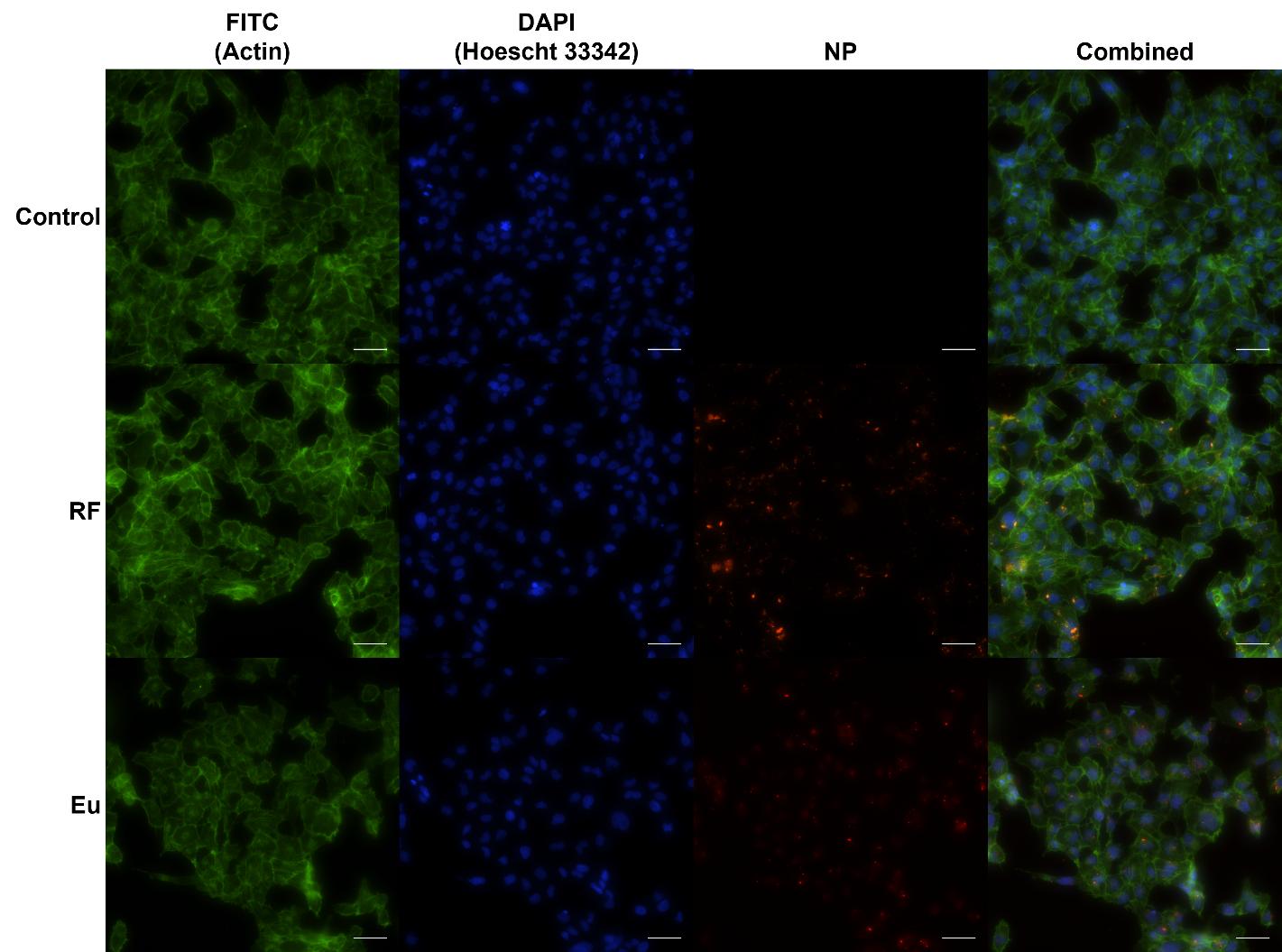


Fig. S16 Internalisation of RF and Eu 100 nm PSNPs after 24-hours of exposure at 7 µg/mL. Cytoskeleton stained with Actin Green (Phalloidin), Nucleus stained with NucBlue (Hoechst 33342). Scale bar = 50 µm. Data is representative of individual replicates tested. N=3


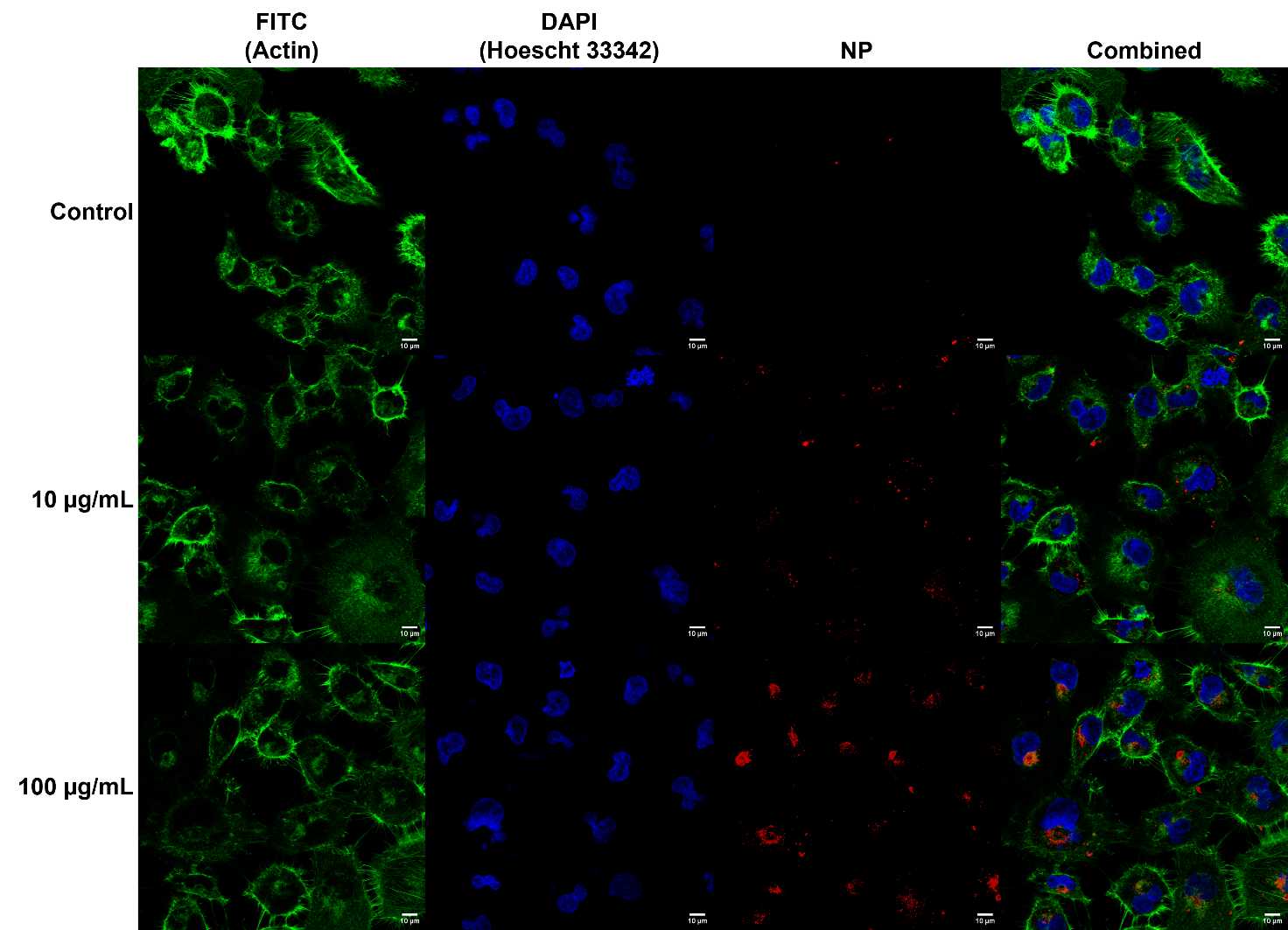


Fig. S17 Internalisation of PE50-B-RF NPs after 24-hours of exposure at 10 µg/mL and 100 µg/mL. Cytoskeleton stained with Actin Green (Phalloidin), Nucleus stained with NucBlue (Hoechst 33342). Scale bar = 10 µm. Data is representative of individual replicates tested. N=3

# References

Ortiz-Tafoya MC, Tecante A. Physicochemical characterization of sodium stearoyl lactylate (SSL), polyoxyethylene sorbitan monolaurate (Tween 20) and κ-carrageenan. Data in Brief. 2018;19:642-50. https://doi.org/https://doi.org/10.1016/j.dib.2018.05.064

Smith B. Infrared Spectroscopy of Polymers X: Polyacrylates. Spectroscopy. 2023;38:10–4.

Smith BC. The Infrared Spectra of Polymers II: Polyethylene. Spectroscopy. 2021a.

Smith BC. The Infrared Spectra of Polymers III: Hydrocarbon Polymers. Spectroscopy. 2021b.

Smith BC. Infrared Spectral Interpretation: A Systematic Approach. 1999.
